# Coronin-1A Links Cytoskeleton Dynamics to TCRαβ-Induced Cell Signaling

**DOI:** 10.1371/journal.pone.0003467

**Published:** 2008-10-21

**Authors:** Bénédicte Mugnier, Béatrice Nal, Christophe Verthuy, Claude Boyer, David Lam, Lionel Chasson, Vincent Nieoullon, Geneviève Chazal, Xiao-Jun Guo, Hai-Tao He, Dominique Rueff-Juy, Andrés Alcover, Pierre Ferrier

**Affiliations:** 1 Centre d'Immunologie de Marseille-Luminy, Université Aix-Marseille, Marseille, France; 2 CNRS, UMR.6102, Marseille, France; 3 Inserm, U.631, Marseille, France; 4 Institut de Biologie du Développement de Marseille, Inserm U.382, Marseille, France; 5 Laboratoire des Interactions Moléculaires et Systèmes Membranaires, Université Aix-Marseille, Marseille, France; 6 Unité de Biologie Cellulaire des Lymphocytes, CNRS URA.1961, Paris, France; 7 Unité d'Immunophysiopathologie Infectieuse, Institut Pasteur, Paris, France; University Paris Sud, France

## Abstract

Actin polymerization plays a critical role in activated T lymphocytes both in regulating T cell receptor (TCR)-induced immunological synapse (IS) formation and signaling. Using gene targeting, we demonstrate that the hematopoietic specific, actin- and Arp2/3 complex-binding protein coronin-1A contributes to both processes. Coronin-1A-deficient mice specifically showed alterations in terminal development and the survival of αβT cells, together with defects in cell activation and cytokine production following TCR triggering. The mutant T cells further displayed excessive accumulation yet reduced dynamics of F-actin and the WASP-Arp2/3 machinery at the IS, correlating with extended cell-cell contact. Cell signaling was also affected with the basal activation of the stress kinases sAPK/JNK1/2; and deficits in TCR-induced Ca^2+^ influx and phosphorylation and degradation of the inhibitor of NF-κB (IκB). Coronin-1A therefore links cytoskeleton plasticity with the functioning of discrete TCR signaling components. This function may be required to adjust TCR responses to selecting ligands accounting in part for the homeostasis defect that impacts αβT cells in coronin-1A deficient mice, with the exclusion of other lympho/hematopoietic lineages.

## Introduction

T lymphocytes are rapidly moving cells dedicated to the recognition of antigenic complexes of peptides bound to MHC molecules on the surface of antigen presenting cells (APCs). The stable interaction between T cell receptor (TCR) and relevant peptide-MHC (pMHC) ligands triggers T cell activation, leading to clonal expansion and differentiation of naïve T cells into effector and/or memory cells. T cell polarization towards the APC occurs during this process, resulting in cytomorphological and molecular changes at the T cell-APC contact zone. This interface, termed the immunological synapse (IS), reorganizes into a highly ordered area where TCRs, co-receptors, adhesion and signaling factors concentrate and segregate into supramolecular activation clusters. Molecular clustering at the IS is though to sustain intracellular signaling necessary for cell activation and/or act as an adaptive controller that attenuates or enhances strong and weak signals, respectively [1]–[3]. Development of T cell precursors through discrete thymic checkpoints and the maintenance of T lymphocytes in the periphery also depend on TCR-ligand interactions. However, the cortical events that may be tied to these processes are less well defined ([4], [3] and references therein).

The evidence that TCR clustering, downstream signaling and T cell proliferation can be abolished by the disruption of actin filaments led to the notion that these structures [referred to as filamentous (F)-actin as opposed to G-actin monomers] are essential for T cell activation [5]. F-actin may be involved in driving molecular movements at the IS and/or providing an organizing scaffold for signaling complexes. Accordingly, many studies have implied important roles for various actin-regulatory proteins in the formation and function of an IS, from the F-actin stabilizer HS-1 to the Wiskott-Aldrich syndrome protein (WASP) and/or homologous suppressor of cyclic AMP repressor (SCAR)/WASP-family verprolin-homologous (WAVE) proteins [6], [7]. The latter proteins, so-called nucleation-promoting factors (NPFs), activate actin-related protein-2/3 (Arp2/3) complex, a cellular device responsible for actin nucleation and branching. Considering the plasticity of the IS, a tightly regulated underlying actin network is expected, with additional cytoskeleton-associated factors operating locally to coordinate and finely tune the actin dynamics in activated T cells.

The coronin family of F-actin- and Arp2/3-binding proteins has been associated with a variety of cytoskeleton-dependent processes in all eukaryotic species examined (*e.g.*, cell migration and morphogenesis; cellular trafficking and cytokinesis [8], [9]). By and large, coronins from different species harbor a common molecular structure comprised of a signature of five consecutive WD40 repeats flanked by conserved N- and C-terminal extensions. In various polypeptides, such as the β subunits of G proteins, clustered WD40 repeats are known to form β propeller structures and mediate protein-protein interactions. Structural analysis of mammalian coronin-1A (see below) indicated that the canonical WD40 repeats and adjacent stretches within the N- and C-terminal extensions organize into a seven-bladed β propeller carrying two potential F-actin binding sites on the top and bottom faces of this core domain respectively [10]. Distal from the C-terminal extension, a unique region differs significantly among coronins in both length and sequence composition. In most coronins, this is followed at the C-terminus by a coiled-coil domain that mediates homo-oligomerization. In the yeast coronin Crn1p, it is this latter area that binds the Arp2/3 complex and inhibits Arp2/3-dependent nucleation of F-actin polymerization [11], [12].

Mammalian genomes encode seven coronin family members (denoted 1A/1B/1C, 2A/2B, 6 and 7) showing distinct patterns of expression across cell types and tissues [9]. Among them, coronin-1A is preferentially expressed in hematopoietic cells. This 57 kDa protein carries a coiled-coil domain of the leucine zipper variety which mediates the formation of homotrimeric complexes [13]. Coronin-1A has been implicated in phagocytosis by neutrophils [14] and macrophages [15], [16], in TCR-mediated activation of T lymphocytes [17], and more generally as a linker between the plasma membrane and actin meshwork, in integrating outside-inside signaling with cytoskeleton dynamics in leukocytes [13]. Similar to Crn1p, coronin-1A was reported to associate with the Arp2/3 complex [18], a property shared with close homologues coronins 1B [19] and 1C [20], implying a common involvement of members in this subgroup in the regulation of Arp2/3-dependent events.

A former study using coronin-1A knockout mice verified that this factor exerts an inhibitory effect on F-actin formation via an Arp2/3-dependent mechanism. This was linked to alterations in both chemokine-mediated cell migration and, through a mitochondrial pathway, lymphocyte homeostasis [21]. However, this work also led to the conclusion that coronin-1A is dispensable for TCR function in T cells. This specific conclusion was challenged by two recent surveys [22], [23], though the latter did question a role for coronin-1A in regulating F-actin dynamics in primary T cells. To learn more about the coronin-1A-dependent control of lymphocyte homeostasis, we also generated coronin-1A null animals. Here, we provide evidence that this factor indeed modulates F-actin and IS dynamics in αβT cells following TCR triggering, with consequences on downstream signaling. We discuss these findings, which reinstate coronin-1A as an entire mediator of TCRαβ-dependent cell development and activation processes, with respect to those from other studies of coronin-1A-null mutants.

## Results

### Generation of Coronin-1A-deficient Mice

To disrupt the mouse *coronin-1A* gene, we employed gene-targeted mutational techniques to delete genomic DNA sequences from the corresponding locus (Materials and Methods S1 and Figure S1A). The deleted region included the translation initiation site for coronin-1A as well as downstream coding sequences up to and including those for domain WD3. To prevent possible neighborhood effects and/or cell alterations caused by expression of the *neo* gene [24], [25], we used *Cre*/*LoxP*-mediated deletion to remove the drug selectable *neo* cassette from the targeted locus (which is scattered with genes of known immunological/metabolic relevance, see [22]). We verified that the intended mutation led to a complete loss of coronin-1A in homozygous animals (henceforth referred to as *Coro-1A^−/−^*; Figure S1A–B). *Coro-1A^−/−^* mice were born at the expected Mendelian frequency displaying no apparent differences from wild-type (WT) and heterozygous (*Coro-1A^+/−^*) littermates in growth, weight, fertility or viability. However, the *Coro-1A^−/−^* mice had substantially fewer cells in their lymph nodes (LNs) and, generally, increased numbers of total thymocytes compared with *Coro-1A^+/−^* and WT controls (Tables 1 and S1). We therefore analyzed the lympho-hematopoietic cell compartments in *Coro-1A^−/−^* animals further.

**Table 1:** Lympho-hematopoietic Cellularity (×10^6^) in Lymph Nodes and Spleen from Wild-Type (WT), *Coro-1A^+/−^* and *Coro-1A^−/−^* Mice.

Lymph Nodes						Spleen						
	Tot. cell	CD8	CD4	Tαβ	Tγδ	Tot. cell	CD8	CD4	B220	Mac.1	NK	DC
WT	24.7±7.3	4.1±1.7	7.0±2.2	12.1±1.9	0.4±0.2	46.6±6.9	7.6±1.0	13.0±1.7	16.6±1.8	4.7±1.2	2.4±0.7	2.2±0.5
*Coro1A^+/−^*	21.0±6.6	4.2±1.9	7.8±3	10.8±2.6	0.3±0.1	44.6±8.2	6.9±1.4	12.0±1.6	15.1±2.1	4.3±1.3	2.6±0.3	2.3±0.7
*Coro-1A^−/−^*	10.0±5.2^a^	1±0.5^a^	1.8±0.8^a^	1.9±0.9^a^	0.5±0.17	44.9±8.3	3.1±1.1^a^	4.3±1.6^a^	26.2±3.2^a^	4.8±1.5	2.6±0.4	3.3±1.0

Data presented are mean values±standard error (95% confidence interval); WT, n = 18; *Coro-1A^+/−^*, n = 15; *Coro-1A* ^−/−^, n = 21.

a Statistically significant difference Student's test, p≤0.005).

### Reduced Peripheral αβT Lymphocytes in Coronin-1A-deficient Mice

In adult mice, coronin-1A is expressed in various hematopoietic cell lineages (Figure S1B). In this context, initial analyses revealed a normal segregation of these lineages in *Coro-1A^−/−^* animals but a reduced density of peripheral T lymphocytes, in general agreement with published data on *coronin-1A* knockout mice [21], [23]. In fact, immunohistological examinations and flow cytometric analyses of various cell lineages in central (thymus and bone marrow) and peripheral (spleen and LNs) lympho-hematopoietic tissues from *Coro-1A^−/−^* mice revealed a sharp reduction of peripheral αβT cells compared to WT controls (especially marked in LNs); with cell numbers in other hematopoietic lineages not severely affected [including macrophages, dendritic cells (DC), NK cells, B lymphocytes, and, most surprisingly, γδT cells] (Figures S2, S3, S4A; summarized in Tables 1 and S1). In the *Coro-1A^−/−^* mice, the residual T cells show signs of increased activation when compared to WT total T cells [*i.e.*, increased percentages of effector/memory (CD62L^lo^/CD44^+^) cells and of CD69^+^, CD24^+^ or CD25^+^ cells], and augmented cell-death (*i.e.*, annexin-V^+^ staining) (Figure S4B). Importantly, mouse reconstitution experiments using cells from the mutant bone marrow also provided evidence for a T (but not B) cell defect, and increased representation of CD69^+^ cells (data not shown) implying a T cell intrinsic function for coronin-1A.

### Coronin-1A Deficiency Impairs T Cell Terminal Differentiation

**Figure 1:**
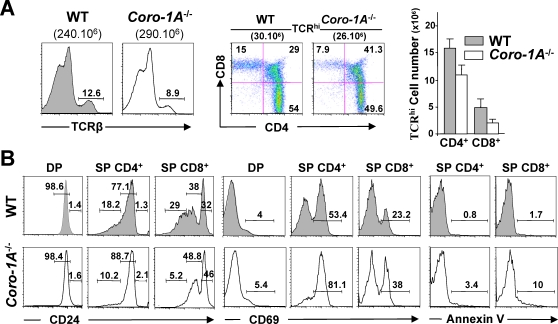
The Coronin-1A Deletion Interferes with Late Development of SP Thymocytes. (A–B) Flow cytometric analysis of thymocytes from WT and *Coro-1A* ^−/−^ mice. In the histograms shown in part (A), total thymocytes were first analyzed for TCRβ cell-surface expression (left); TCRβ high (TCR^hi^)-expressing cells were then analyzed for CD4 and CD8 surface expression (middle), and cell numbers found in these experiments are presented in the graphs on the right (four mice of each type were analyzed). (B) Analysis of CD24, CD69, and annexin-V surface staining in thymocyte subsets. Percentage of positive-scoring cells within the CD24^hi^, CD24^int^ or CD24^lo^ windows (left), and the CD69^+^ (middle) or annexin V^+^ (right) windows, are shown (representative results from four separate experiments).

Although T cell development in *Coro-1A^−/−^* mice did not seem grossly affected, several findings prompted us to reconsider the possible outcome of an altered developmental event. Thymic cell count was generally enhanced in *Coro-1A^−/−^* mice due to an increase in DP cells, despite no detectable change in cell proliferation. Yet, SP cells appeared proportionally reduced (Figure S2B; Table S1). Deeper examination using age-matched controls revealed a reduction in percentages and absolute cell numbers of TCRαβ high expressors (TCR^hi^) among coronin-1A-deficient CD8^+^ and, to a lesser extent, CD4^+^ thymocytes (Figure 1A). Also, *Coro-1A^−/−^* SP thymocytes had reduced proportions of mature (CD24^lo^) cells and, conversely, enhanced proportions of CD69^+^ and annexin V^+^ cells (Figure 1B). Thus, compared to WT controls, a larger fraction of *Coro-1A* ^−/−^ SP cells displays less-mature phenotypic traits (TCRβ^int^, CD24^int/hi^ and/or CD69^+^) and enhanced cell-death, suggesting that the critical function(s) of coronin-1A in maintaining T-cell homeostasis overlaps the end window of αβT cell development.

In separate analyses (to be submitted elsewhere), we tested the hypothesis of an irregular selection event to further explain the paradoxical observation of DP-cell accumulation in mutant mice, and lower yield of mature SP cells. Notably, analysis of CD8^+^ SP cells for which the TCR repertoire variability had been reduced by expression of the MHC Class I-restricted H-Y or P14 TCR transgene (tg) [26], [27] inferred an altered TCRαβ selection in the mutant mice (data not shown). This was especially marked for those bearing the P14 TCR which when compared to the H-Y TCR binds its self-pMHC ligand with a higher affinity [28]. In the periphery, absolute numbers and percentages of αβT cells were reduced in *Coro-1A^−/−^*×tg mice, as expected; with a particularly diminished generation of P14 tg-bearing cells (Table 2). Collectively, our studies unveil an impact of coronin-1A on the late development of αβT cells. Several features of coronin-1A-null SP cells (*e.g.*, altered CD4/CD8 ratio, preferential deletion of high-avidity TCR-bearing cells) evoke a TCR-dependent process and the prospect that, at this stage, coronin-1A contributes to modulate the strength of TCR downstream signaling.

**Table 2:** LN Cell Numbers (×10^6^) in Conventional (+/+) and Coronin-1A-deficient (−/−) H-Y or P14 TCR Transgenic (tg) Mice.

	Tot. Cells	Tot. CD4^+^	Tot. CD8^+^	CD8^+^ tg TCR^+^
+/+ (H-Y tg)	50±2	14±2	6±1	2±0.3
−/− (H-Y tg)	26±3	8±4	2.8±0.2	1±0.1
+/+ (P14 tg)	22±6	8.5±4	12.5±2	11±2
−/− (P14 tg)	13±0.1	1.4±0.2	2.3±0.2	1.3±0.3

Data presented are mean values±standard error (95% confidence interval); n = 3 for all types of mice; H-Y and P14 tg TCR-bearing cells were detected by the specific T3.70 mAb and the anti-Vα2 mAb, respectively.

### Reduced Accumulation of Coronin-1A-deficient T Cells in Response to TCR Stimulation

**Figure 2:**
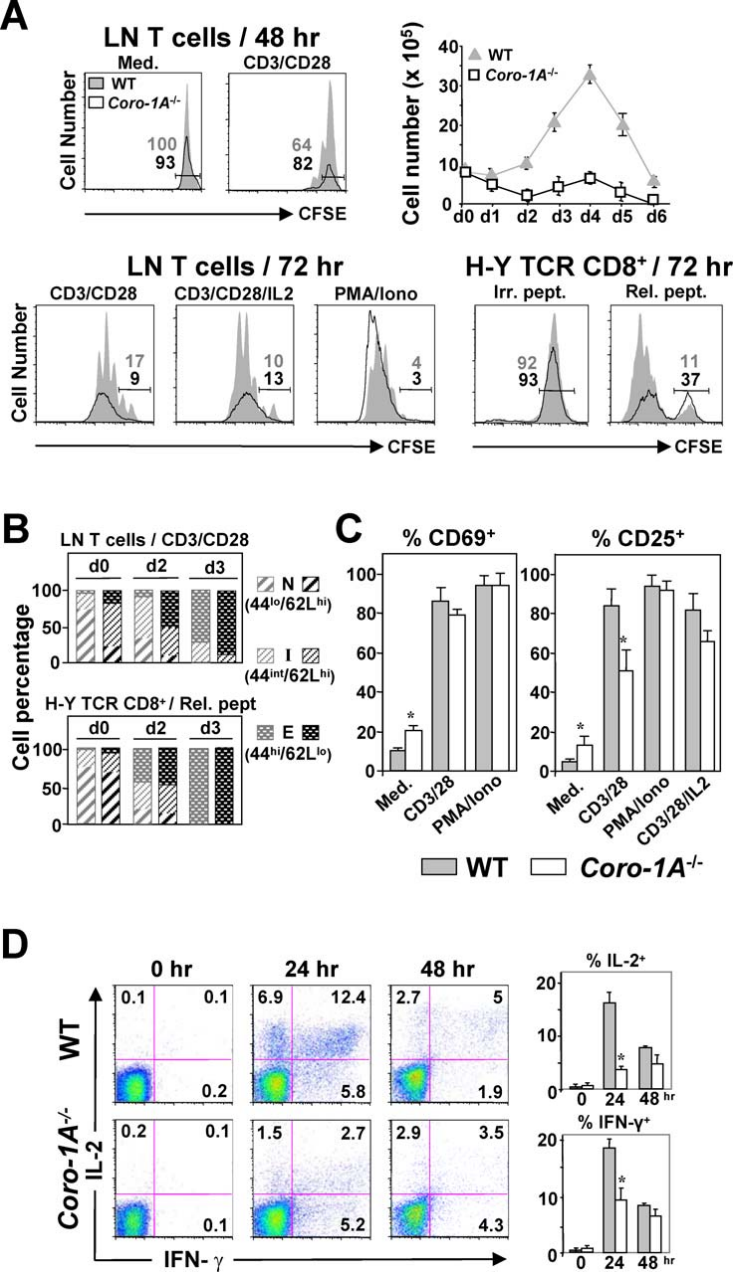
Analysis of TCR-induced Activation of Coronin-1A-deficient T Cells. (A–B) T cell proliferation. (A) LN T cells from coronin-1A-deficient (open areas) and control (WT or conventional tg; grey areas) mice were labeled with CFSE, and cultured in the indicated conditions. Irr./Rel. pept.: irrelevant or relevant (agonist) peptide, respectively. At the indicated time, cells were stained for annexin V and CFSE dilution was monitored by FACS on annexin V^−^ cells. Ordinates of the CFSE graphs indicate the numbers of living (annexin V^−^) cells recovered in each type of mice; percentages of non-divided cells are indicated. CD3/CD28-stimulated cell numbers were also assessed by trypan blue exclusion from day (d)0 to d6 (upper diagram). The data are representative of four separate experiments. (B) Surface expression of CD44 and CD62L were measured by FACS, at the indicated time (d0, d2 and d3), in the experiments of CD3/CD28 or H-Y TCR tg stimulation shown in (A). Cells were classified as naïve (N; CD44^lo^CD62L^hi^), intermediate (I; CD44^int^CD62L^hi^), or effector (E; CD44^hi^CD62L^lo^) T cells. (C) Percentages of CD69 and CD25-surface-expressing cells were determined by FACS after, respectively, 24 and 48 hr of culture in the indicated conditions (three mice of each type were analyzed). (D) Analysis of IL-2 and IFN-γ intracellular production. Purified LN T cells were stimulated using anti-CD3/-CD28 mAbs for the indicated periods of time, then re-stimulated for 4 hr in the presence of brefeldin A. Percentages of IL-2 and IFN-γ producing cells were determined by FACS. A representative experiment (out of three), is shown on the left. *P<0.01 for all graphs.

To explore this hypothesis further, we analyzed the activation events induced in coronin-1A-null T cells upon TCR triggering. In this condition, T cells normally undergo a rapid expansion coupled to the acquisition of effector functions. Using carboxyfluorescein diacetate succinimidyl esther (CFSE) dilution, we first assessed the proliferative capacity and expansion of *Coro-1A* ^−/−^ *vs.* WT LN T cells following CD3/CD28 stimulation, focusing on living (annexin V^−^) cells. As shown in Figure 2A, the WT but not the mutant T cells underwent initial cell-division readily countable from day 2 [top left; 1–2 cycle(s)]. Live-cell counts indicated stable numbers of WT cells during the first 48-hrs, as expected, while those of *Coro-1A* ^−/−^ cells steadily dropped (top right). Cell expansion then prevailed among both WT and mutant cells (top right and bottom left). Due to ongoing cell death however (and likely a delayed entry into first division), the integrated areas of CFSE dilution were consistently smaller for the *Coro-1A^−/−^* T cells, indicating in the end a 2–3 fold reduced accumulation. Exogenous supply with the interleukin (IL)-2 cytokine showed little improvement in these *Coro-1A* ^−/−^ T cell patterns. Yet, treatment with phorbol esther PMA and Ca^2+^ ionophore ionomycin (which bypasses TCR cell-surface triggering) yielded a cell accretion comparable to WT controls, arguing for both a TCR activation disorder in *Coro-1A^−/−^* T cells and the conservation of distal signaling [*i.e.*, at the level and/or downstream of protein kinase C (PKC)]. These results were not due to a decreased expression of CD3 or CD28 on the surface of the mutant T cells (not depicted). [^3^H]thymidine incorporation studies agreed with the above findings (Figure S5A). We observed similar profiles on analysis of coronin-1A-null, CD8^+^ T3.70^+^ T cells when mixed with agonist Ag-pulsed APCs; possibly marked by less divisions relative to CD3/CD28-stimulated *Coro-1A^−/−^* T cells (Figure 2A, bottom right). Of note, in the absence of intentional stimulation, mutant T cells expressing the H-Y TCR mainly displayed a naïve phenotype similar to CD8^+^ T3.70^+^ T cells (Figure 2B, d0 profiles; also see [29]), arguing for the reduced expansion of coronin-1A null T cells mainly depending on this factor's ablation rather than on their resulting phenotype. In allusion to the intended hypothesis, it is worth stating that a recent study has enlightened the importance of TCR-ligand affinity (hence inherent signal intensity) on individual kinetic features of T cell proliferation, particularly the entry time into first division and cell survival during proliferation, and not the consecutive division rate [30], forecasting precisely the cell division profiles reported here for the *Coro-1A* ^−/−^ T cells.

### Coronin-1A is Required for TCR-induced Production of the IL-2 Cytokine

TCR stimulation eventually results in the transactivation of many gene products, including the early (CD69) and later (CD25) activation antigens, as well as cytokines such as IL-2 and interferon (IFN)-γ. Though enriched in CD69 or CD25 surface-expressing cells at a basal state, *Coro-1A^−/−^* LN T cells differentially improved their expression patterns following CD3/CD28 engagement. At 48 hr in particular, CD25^+^ cells were significantly less represented among TCR-triggered mutant T cells compared to WT controls (Figure 2C) and showed lower expression for this marker (Figure S5B). PMA/ionomycin or the supply of IL-2 attenuated these differences. In separate FACS (intracellular) studies, we found a smaller percentage of activated *Coro-1A^−/−^* T cells synthesizing IL-2 and, to a lesser degree, IFN-γ compared to WT controls (respectively, 4±1% *vs.* 17±2.5% and 9±2.5% *vs.* 18±1.5% of producers after 24 hr of stimulation; Figure 2D). As the FACS analysis only considered cells with scatter signals typical of viability, the defects are unlikely to simply reflect enhanced death of the mutant T cells. The weak IL-2 (and reduced CD25/IL-2Rα chain) response may affect the survival of the dividing T cells [30], thus rationalizing the data presented above. Interestingly, despite a lower representation of IL-2/IFN-γ double producers among *Coro-1A^−/−^* stimulated T cells, that of cells producing IFN-γ only was less affected (Figure 2D). A hierarchical acquisition of effector functions dependent on factors associated with priming conditions has been documented (*e.g.*, in normal T cells, the activation threshold to produce IFN-γ is lower than that required for entry into the cell-cycle or to secrete IL-2 [31], [32]). This raises the possibility that the impairment of cellular functions seen in coronin-1A deficient T* cells may be more specific to those requiring the highest activation thresholds.* Overall, the reduction in TCR-induced cell accumulation of, and cytokine production by, coronin-1A-null T cells could be related to a general dysfunction in the total population, perhaps with the presence of subsets of coronin-1A-highly and -less dependent T cells.

### Coronin-1A Interferes with F-actin Dynamics and Redistribution at the T/APC Contact

**Figure 3:**
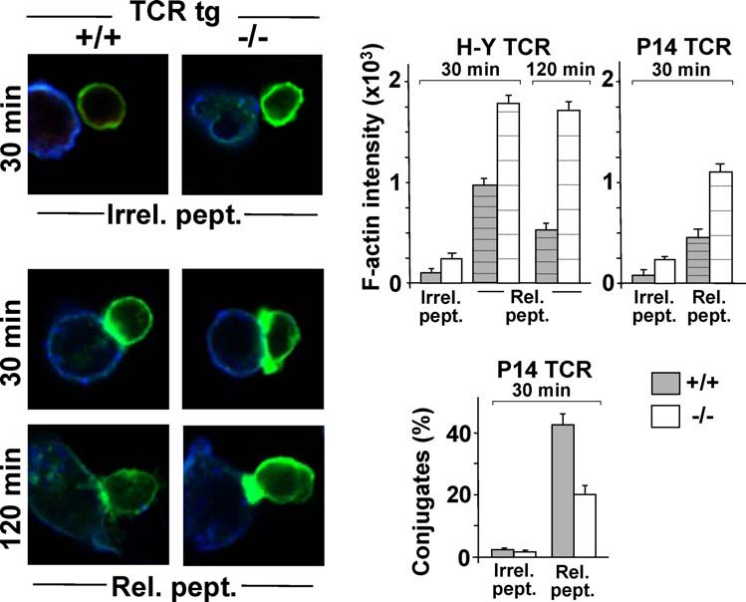
TCR-induced Reorganization of F-actin in Coronin-1A-deficient T Cells. Purified CD8^+^ LN T cells from conventional TCR tg (+/+) and *Coro-1A^−/−^*×TCR tg (−/−) mice were mixed with Cy5-labelled, peptide-pulsed RMA-S cells (blue) (Irr./Rel. pept.: irrelevant or relevant peptide, respectively). At the indicated time, the cells were fixed, permeabilized and stained for F-actin (PhalloidinA-488, green). Representative images from H-Y TCR transgenic mice are shown on the left. The upper graphs indicate mean values of fluorescence intensity at T-cell/APC interfaces for each H-Y or P14 tg mouse model (assessed from the unprocessed images using Zeiss LSM 510 software). Percentages of cell conjugates (as determined by cell counting) using the P14 tg model are shown at the bottom (results from three experiments).

The latter results differ from those obtained by Föger et al. [21] asserting that coronin-1A was dispensable for TCR function in T cells. This prompted us to re-assess a possible interference of coronin-1A deficiency on F-actin redistribution events in response to TCR triggering. Using the H-Y and P14 TCR tg mouse models and confocal microscopy, we performed an analysis of phalloidin-stained F-actin in purified CD8^+^ T cells from conventional tg or compound *Coro-1A^−/−^*×tg mice stimulated with agonist peptide-loaded APCs. As controls, we used CD8^+^ T cells mixed with APCs pulsed with an irrelevant peptide. As shown in Figure 3, coronin-1A-deficient tg T cells displayed higher amounts of F-actin compared to their conventional tg homologues, even following mock stimulation. When challenged with cognate ligands, the former cells exhibited F-actin-rich membrane protrusions at the interface with the APC that were larger than those seen in control cells and also lasted for a longer period of time. Computer-assisted image quantification (top graphs) implied that the rate of F-actin redistribution to the T/APC contact (hence cytoskeleton plasticity) was generally lower in the mutant *vs.* conventional tg T cells (despite higher steady-state levels), and lower in P14 *vs.* H-Y TCR-expressing cells (4.5× *vs.* 7.4× at 30 min). In parallel, we observed a reduced overall fraction of agonist peptide-induced APC/T-cell conjugates in coronin-1A-null T cells [determined by cell-counting (bottom graph) and confirmed by flow cytometry (data not shown)], especially when using the P14 model. These results argue for an impact of coronin-1A in modulating F-actin and IS plasticity in response to TCR triggering. Consistently, we also observed altered responses of F-actin polymerization following CD3 stimulation in FACS analyses of *Coro-1A^−/−^ vs.* WT T cells (reduced amplitude, though initiating from a higher steady-state; but longer time-span), a behavior that correlated with enhanced adherence in cell-spreading assays (Figure S6A–B).

### Accumulation of the Arp2/3complex/WASP Machinery at the APC Contact in Coronin-1A-null T Cells

**Figure 4:**
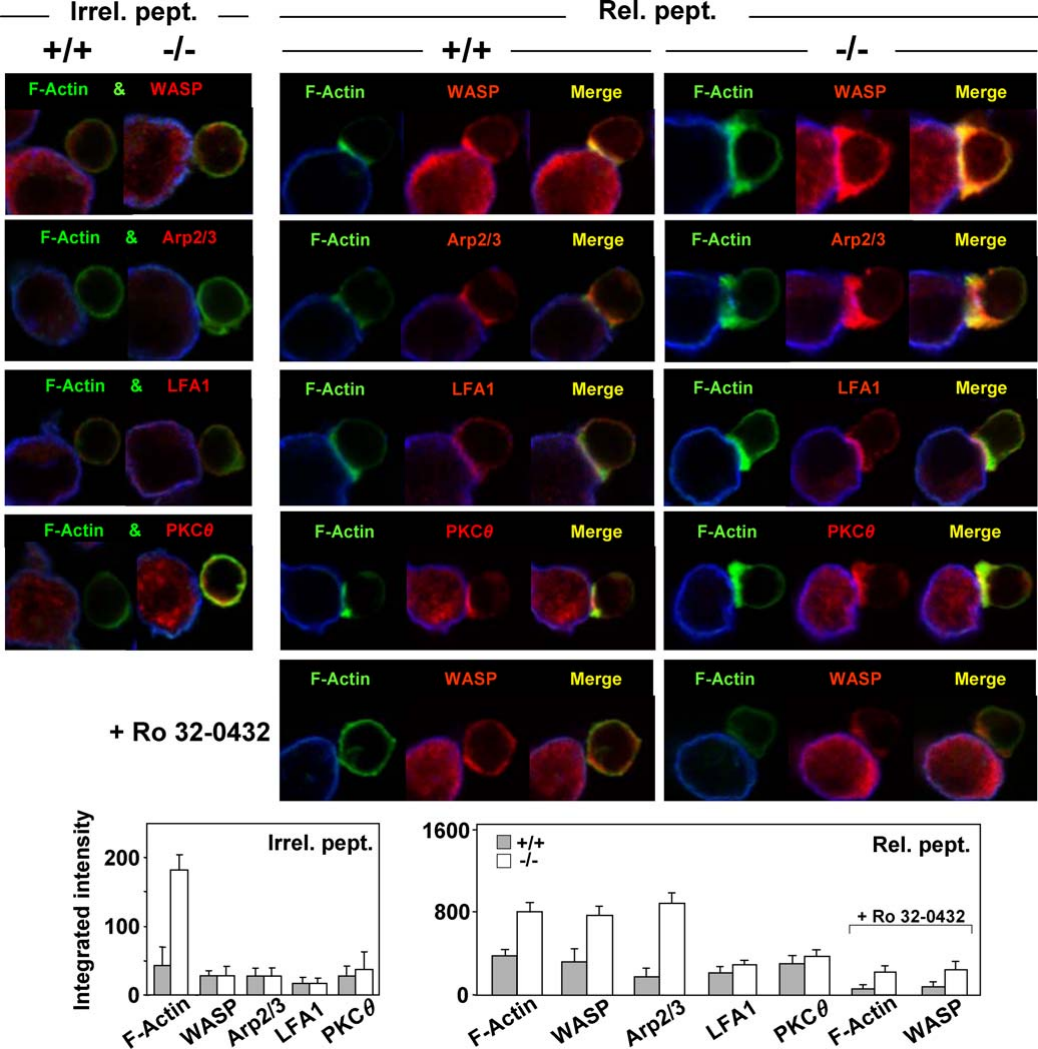
Ag-induced Accumulation of the Arp2/3/WASp Machinery at the APC Interface of Coronin-1A-deficient T Cells. Confocal analyses were performed as described in Figure 3 using purified CD8^+^ T cells from P14 TCR tg (+/+) and *Coro-1A^−/−^*×P14 TCR tg (−/−) mice (contact time of 30 min). Fixed cells were incubated with anti-LFA-1 (non-permeabilized cells) or anti-WASP, -p34-Arc/ARPC2 (Arp2/3 complex subunit), or -PKC*θ* (permeabilized cells) Abs, then with secondary, biotinylated Abs and streptavidin A546 (red). Phalloidin A-488 (green) labeling of F-actin was then performed. F-actin and WASP accumulation were also examined in the presence of the pan-PKC inhibitor Ro 32-0432. Statistical analysis of factor polarization to the T-cell/APC contact zone (bottom graphs) was performed based on computer-assisted quantification of integrated intensities [Area×Mean Fluorescence Intensity/µm^2^]. Values are from three experiments (≥50 conjugates were scored in each experiment).

The molecular control of F-actin assembly and disassembly upon TCR stimulation is highly regulated and coordinated. The Arp2/3 complex is considered a critical (though not exclusive) regulator of polarized F-actin reorganization at the IS [33]. Structural studies have shown that this complex exists in a distribution of conformations, with the WASP NPF and coronin inhibitor contacting distinct regions of the complex, thereby exerting opposing effects to displace the equilibrium towards respectively a closed (active) or open (inactive) form [11]. This inhibitory function of coronin factors to counteract the activity of NPFs may well explain the basal accumulation of F-actin that we (*e.g.*, Figure 3) and others [21], [22] have observed in non-stimulated coronin-1A null T cells and also, remarkably, in coronin-1A null B cells* ([22]; our unpublished data). Since the homeostasis defect in the mutant mice prevails for T cells also displaying altered dynamics in their ability to modulate F-actin reorganization at the IS, we next examined the WASP and Arp2/3 complex reorientation towards APCs. Confocal assays demonstrated a spectacular increase in these two factors in coronin-1A null T cells at their APC interface, that superimposed with F-actin in a quantitatively differential mode compared to conventional (coronin-1A^+/+^) controls (Figures 4 and S6C). In parallel, PKC*θ* and the lymphocyte function-associated antigen (LFA)-1 integrin, two well-established components of the IS, displayed roughly equal profiles in both types of cells. The accumulation of Arp2/3 complexes and WASP to the IS of mutant T cells suggests a role of coronin-1A in adjusting the balance of F-actin branched regulators at this site after TCR triggering. The observations of WASP but not of PKC*θ* accumulation are particularly intriguing as these two factors were recently reported to exert opposing (respectively negative and positive) effects on IS relocation, a process also correlated with greater IL2 production [34]. In this context, it is notable that treatment with the pan-PKC inhibitor Ro 32-0432, which impairs T-cell activation [35], did not totally abolish (though greatly reduced) F-actin and WASP polarizations towards the APCs in coronin-1A-null T cells contrary to the corresponding controls (Figure 4, lower panels). This supports an impact of WASP accumulation on exaggerated IS stability once the coronin-1A function is impaired.

### Signal Transduction in Coronin-1-A-deficient T Cells

**Figure 5:**
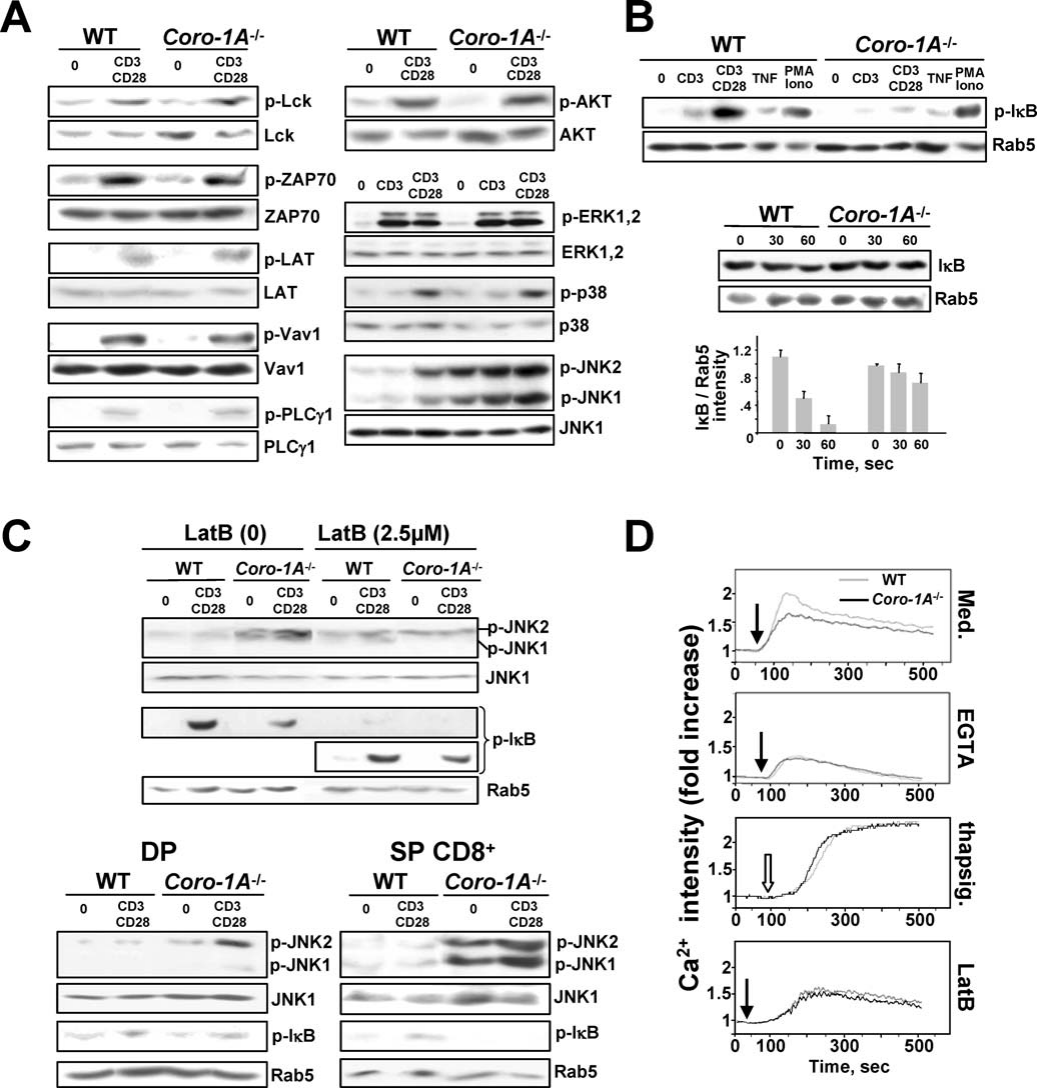
Signal Transduction in Coronin-1A-deficient T Cells. (A–C) Phosphorylation of the indicated signaling molecules was tested by Western blotting of protein lysates from thymocytes (5×10^6^ cells) of WT or *Coro-1A^−/−^* mice using appropriate mAbs and the indicated stimulation conditions. Blots were reprobed for the non-phosphorylated protein, or for Rab5, as a loading control. (B) IκB degradation was assessed in total thymocytes activated with anti-CD3/-CD28 mAbs for 30 min. (C) Top panels: JNK or IκB phosphorylation in CD3/CD28-stimulated WT and *Coro-1A^−/−^* thymocytes pre-treated or not with LatB (0 or 2.5 µM); bottom panels: same analysis from purified DP and CD8^+^ SP thymocytes. All membrane exposures were for 2 min, except for the analysis of IκB phosphorylation following LatB treatment for which a longer exposure is also shown (20 min/middle panel of this experiment). (D) Analysis of Ca^2+^ responses using indo-1AM imaging and flow cytometry. Ca^2+^ measurements were performed using single cell fluorescence ratio of freshly isolated WT and *Coro-1A* ^−/−^ thymocytes incubated in Ca^2+^-containing medium in the absence or presence of EGTA or LatB, then stimulated by the addition of an anti-CD3ε Ab (closed arrows) or thapsigargin (thapsig.; open arrow). Fluorescence emission at 526 nm was recorded at 30 sec intervals. The curve, in ‘fold increase’, represents the medium fluorescence intensity of an activated sample at a discrete time compared to that of the corresponding sample before activation (baseline value of 1). All data are representative from at least three experiments.

To further unravel the biochemical pathways responsible for the functional defects associated with an absence of coronin-1A, we investigated TCR-induced signaling in lymphoid T cells. Due to the scarcity of peripheral T lymphocytes in the *coro-1A* ^−/−^ mice, we mainly used thymic-cell samples to perform these experiments. Using Western blots of nuclear extracts and dedicated antibodies, we first compared tyrosine phosphorylation induced in WT and *coro-1A* ^−/−^ thymocytes by CD3 or CD3/CD28 stimulation, and found no alteration at the level of proximal signaling factors p56^Lck^, ZAP-70, LAT, Vav-1 and PLCγ1; or more distal MAP kinases (MAPK) ERK and p38 (Figures 5A and S7A). CD3/CD28-induced phosphorylation of Akt also implied normal regulation of PI3-kinase pathways. However, *Coro-1A* ^−/−^ samples displayed steady-state phosphorylation of sAPK/JNK1/2 (Figure 5A, bottom right) implying a constitutive activation of the JNK pathway. Furthermore, we noted a drop in IκB (inhibitor of NF-κB) phosphorylation in stimulated thymocytes (and peripheral T cells) from *Coro-1A^−/−^* mice (Figures 5B and S7B). This defect, more marked with CD28 co-stimulation, came with a delay in IκB degradation, as expected. The lack of change in IκB phosphorylation in these cells following TNFα receptor triggering or PMA/ionomycin treatment (Figure 5B) implies that coronin-1A deletion specifically affects the NF-κB pathway downstream of the TCR. JNK1/2 phophorylation displayed sensitivity to latrunculin B (LatB; Figure 5C top panels), a compound which induces F-actin depolymerization. LatB also seemed to differentially down-modulate IκB phosphorylation in WT *vs. Coro-1A* ^−/−^ T cells (less pronounced in the latter; see the residual footprints following longer exposure of the transferred membrane), indicating a possible link between TCR-induced alterations in F-actin meshing and NF-κB signaling in coronin-1A-null T cells. We observed a stronger aberration of both JNK1/2 and IκB phosphorylation in SP cells compared to DP thymocytes (Figure 5C, bottom panels), correlating with a clear variation in F-actin intensities between WT and *Coro-1A^−/−^* T cells from the SP stage onwards (Figure S7C). Finally, cell calcium (Ca^2+^) measurements by flow cytometry in both thymocytes and peripheral T cells provided evidence that coronin-1A deletion also leads to a TCR-induced defect in the mobilization of extracellular Ca^2+^ (rather than Ca^2+^ from intracellular stores as assessed using the chelating agent EGTA or the inhibitor of the endoplasmic reticulum Ca^2+^ ATPase thapsigargin); again, the Ca^2+^ response in WT *vs. Coro-1A^−/−^* thymocytes was differentially sensitive to LatB, the divergence first becoming substantial in SP cells (Figure 5D and data not shown). Collectively, these signaling profiles of *Coro-1A^−/−^* αβT cells provide a framework to link distinctive aberrations in F-actin/IS dynamics, T cell activation and survival/homeostasis in coronin-1A knockout mice (with the paradoxical situation in which F-acting/IS exaggerated stability would in the end result in reduced TCR signal transduction along the NF-κB and Ca^2+^ pathways). Furthermore, they endorse a functional role for coronin-1A in the late phase of αβT cell development.

## Discussion

Genetic ablation of coronin-1A was previously reported to reduce T lymphocyte mobility and viability in connection with this factor's general function of inhibiting Arp2/3-induced nucleation of F-actin polymerization [21]. From there, enhanced cell death was linked to mitochondrial membrane depolarization, an event leading to the release of reactive oxygen species detrimental to cell well-being and longevity [36], with no further thought towards cell specificity [21]. Here, we focused on how coronin-1A deletion severely affects the homeostasis of αβT cells, with the exclusion of other lympho/hematopoietic lineages. In addition to the signaling flaws underlined above, our data document (*i*) an “inhibitory wedge” in αβT cell late development; (*ii*) defects in TCR-triggered cell-division/expansion and IL-2 production; and (*iii*) aberrations in F-actin dynamics and the loading of the nucleation machinery at the IS. In this context, the observations of exaggerated cell death *and* defects in TCR-induced signaling may represent two facets of the same underlying phenomenon, *i.e.*, the alteration of Arp2/3-mediated actin branched nucleation. Several additional findings support a tight connection between these various processes in αβT cells. In a T cell line, actin stabilization enhances apoptosis induced by IL-2 deprivation [37]; whereas a NPF such as WASP is positioned to play a regulatory role in balancing IS breaking and the control of T cell priming and IL2 synthesis [34]. Thus, the mechanism responsible for T-cell deficiency in coronin-1A null mice may be more reliant on TCR-mediated activation events than initially thought. While this paper was in preparation, defects in TCR-induced T cell proliferation and activation have also been reported in two distinct studies of coronin-1A null mice [22], [23] (discussed further below).

Coronin-1A-deficient T cells present a number of signaling aberrations that set out in a developmental manner from the SP thymic cell stage onwards, correlating with excessive F-actin assembly and deletion of TCR^hi^-expressing cells. One such aberration includes the basal phosphorylation of JNK1/2, *ie.*, stress inducible MAPKs that regulate numerous physiological and pathological processes [38], [39]. LatB sensitivity of JNK phosphorylation in *Coro-1A^−/−^* T cells supports the idea that this shift depends on cytoskeletal deformation. However, a backward effect of activated JNKs on cytoskeleton reorganization [40] cannot be excluded. Indeed JNK has been implicated in mediating cell-survival (and, for JNK2, also cell activation) or cell-death depending on the state and time course of activation. This alteration, either separately or in combination with TCR-induced signaling defects [*e.g.*, ablation of an antagonist (NF-κB/calcineurin Aβ) prosurvival activity [41], [42]; see below], may therefore contribute to the complex phenotypic traits of αβT cells observed in coronin-1A-null mice, eventually promoting apoptosis via sustained activation.

Blocks on TCR-induced IκB turnover and Ca^2+^ entry most likely also take place in relation to F-actin altered dynamics in coronin-1A deficient T cells. Each process normally causes the activation of signaling pathways (NF-κB and calcineurin/NFAT pathways) controlling gene expression programs leading to thymic selection and T cell survival, IS formation, clonal expansion and secretion of effector cytokines [43], [44]. This semeiology is consistent with alterations of the two processes adding to the *Coro-1A^−/−^* phenotype, an assumption also supported by data mapping the signaling defects upstream or at the level of PKC activation. Intriguingly, both routes require membrane recruitment of multimolecular operating devices (referred to as the NF-κB signaling complex and calcium release activated calcium (CRAC) channels, respectively [45], [46]). Such spatially and temporally regulated relocalization events may be affected by alterations in F-actin dynamics. Indeed, a negative role of the cytoskeleton on either NF-κB [47], [48] or Ca^2+^ signaling [49] has been evoked. Initial attempts to test this hypothesis in coronin-1A null T cells revealed no apparent fault in the recruitment of PKCθ and the NF-κB signaling complex components Carma1, Bcl10 and IKKβ at the APC contact area (Figures 4 and S6D); with proximal events in TCR signaling also appearing unaltered (Figure 5A). Other ways in which coronin-1A may impinge on the orchestration of TCR-induced IκB degradation and, potentially, on functional properties of the CRAC channel regulators (STIM and ORAI) of Ca^2+^ influx, remain to be determined.

The collapse in peripheral αβT cells in *Coro-1A^−/−^* mice is likely to also reflect an impairment in the trafficking ability of the mutant T cells as originally proposed [21]. Using both *in vivo* and *in vitro* assays [homing aptitude to secondary lymphoid organs of CFSE-labeled cells; transwell migration of T and B cells induced by the SDF-1α chemokine], we confirmed that T lymphocytes from *Coro-1A* ^−/−^ mice display a cell-autonomous defect in their migratory capacity (not shown). In exploring this defect, we additionally found that *Coro-1A* ^−/−^ T cells present a lower frequency of SDF-1α-induced ruffling compared to WT T cells (by ∼2 fold; Figure S8 and Movie S1), reminiscent of the half-speed velocity displayed by coronin-null cells in *D. discoideum* [50]; and consistent with an ancestral input of coronin factors into cytoskeletal remodeling activities dependant on branched-actin nucleators [8], [9]. Another input relevant to cell trafficking alteration may involve the aberrant expression of an activation marker(s) (*e.g.*, CD69) and a potential defect in lymphocyte egress from lymphoid organs [51], within a broader picture of multi-level biological impacts of the coronin-1A deletion.

Images of prolonged APC/T cell contact and reduced conjugate numbers strongly suggest that coronin-1A-deficiency impedes IS dynamics and, possibly, discrete T cell functions. In support of this, we also observed a reduced cytolysis of agonist peptide-loaded cells by TCR tg/coronin-1A-null CD8^+^ T cells (evidenced by ^51^Cr-release assays) despite the acquisition of a lytic function (as assessed by measuring intracellular granzyme B) (our unpublished data). Intriguingly, defects in TCR-induced F-actin/IS dynamics were not found in other studies of *coro1A* knockout mice [21], [23]. Moreover, when assessed, TCR signaling gave a very different picture of a defect in Ca^2+^ mobilization from intracellular stores and reduced ERK1/2 activation [23]. Differences between the various studies of coronin-1A null T cells regarding TCR-mediated activation might be explained by the use of distinct genetic backgrounds and/or distinct cell systems of analysis (transgenic *vs.* polyclonal, CD4^+^ *vs.* CD8^+^ T cells; with dissimilar signaling potencies, [21]–[23], [52] and see below). However, discrepancies in actin dynamics and TCR signaling between data from the knockout reports [21], [23] and those detailed here could also result from variations in gene targeting strategies (*e.g*, the selectable cassette was left at the targeted locus in the previous two studies thus exposing to “*neo* gene” adverse effects). In this context, it is of note that the *Coro-1A^−/−^* T cell phenotypes in relation to Ca^2+^ fluxes, amounts of cellular F-actin, and rescue of the cell-activation block by exposure to PMA/Iono, match those reported in mice carrying a nonsense (spontaneous/not selectable) mutation in the *coro1A* gene [22].

Several additional observations suggest that the severity of phenotypic traits of coronin-1A deficient T cells varies proportionally to the affinity of TCR-ligand interaction, consistent with the view that coronin-1A impacts on TCR signaling potential. One salient aspect is the marked drop in Vα2^+^ *vs.* T3.70^+^ T cells from *Coro-1A^−/−^*×P14/H-Y tg mice (Table 2), as CD8^+^ P14^+^ cells usually display relatively fair homeostatic fitness in a T-cell depleted situation, higher than that of CD8^+^ H-Y^+^ cells [53], [54]. This parallels a similar bias in the counter-selection of Vα2^+^ *vs.* T3.70^+^ coronin-1A null thymocytes (our unpublished data) and a recognized disparity in affinity for self-pMHC ligands between the corresponding TCRs [28]. We envisage two possible ways of interpreting these data and the alleged role of coronin-1A in regulating TCR-dependant αβT cell development and survival. Coronin-1A may act to tamper the signaling potency of TCRs showing relatively high affinity for self-pMHC ligands (such as P14) so that positive selection would, in these cases, be converted into a negative selection in the mutant T cells. However, such a role of coronin-1A, implying increased TCR signaling capacity when deleted, is difficult to match with the frequently reduced outcomes observed in *Coro-1A^−/−^* thymocytes/T cells following TCR-triggering and the fact that negative selection was not enhanced in the absence of this factor (our unpublished results). Alternatively, coronin-1A deletion may instead lead to impaired positive selection and/or homeostatic survival of T cells, more marked in the situation of high affinity self-pMHC/TCR interaction ([55] and references therein). These divergent possibilities are being assessed.

A basic function of coronin-1A in counterbalancing F-actin branched nucleation coupled to TCR engagement, may thus account in part for the homeostasis defect which overwhelms the *Coro-1A* ^−/−^ phenotype. However, the question remains as to how this then leads to the specific disappearance of αβT cells in such a precise temporal manner. Perhaps, in early DN/DP developing T cells (and the γδT and B lineages), the expression of a close homologue(s) sharing similar functional properties (coronin-1B/-1C; data not depicted) compensates for the coronin-1A deficiency. However, the lack of redundancy in αβT cell late development and beyond provides evidence for a possible exclusive role of coronin-1A in the proper tuning of F-actin dynamics at these later stages. Cells differentiated in this lineage may be extremely sensitive to an alteration of cytoskeleton plasticity and effects on TCR downstream signaling. Terminal development of αβ T cells, still an incompletely understood process, is marked by events such as the completion of positive selection and (co)receptor tuning, both requiring the recurrent engagement of their TCR (*e.g.*, [56], [57]). Notably at this point, functional interplays and signaling properties wired to the TCR change, involving ligand selective modulations in TCR sensitivity [58], [59]. The cytoskeleton may have a so-far underestimated role in securing these regulatory programs. Recently, unforeseen IS regulatory functions have been uncovered, including the modulation of Ag stimulatory potency [60] and cell fate outcome [61]. Likewise, a coronin-1A-dependent readjusting of F-actin dynamics in proportion to the strength of the selecting signal may be required as T cells eventually acquire a functional immune repertoire. A failure to attend to these adjustments may, from this critical point in time onwards, irreversibly compromise αβT cell function and survival.

## Materials and Methods

### Mice

All mice used in this study were housed under specific pathogen-free conditions and handled in accordance with French and European directives. All mice were maintained on a C57BL/6J genetic background. The generation of coronin-1A-deficient animals, including DNA cloning procedures, is detailed in the supplemental ‘Materials and Methods’ section.

### Flow Cytometry, Reagents and Cell Purification

Single-cell suspensions of lymphocytes/hematopoietic cells were prepared and stained with antibodies following standard procedures. LN cells were prepared from mesenteric lymph nodes. Single-cell suspensions were analyzed on a FACScan (Becton Dickinson, Mountain View, CA). Phycoerythrin (PE)-, Cy-Chrome C (CyC)-, allophycocyanin (APC)- and peridinin-chlorophyll protein (PerCP)-cyanine (Cy) 5.5 conjugated mAbs against CD8 (53-6.7), CD4 (L3T4 RM4-5), CD44 (Pgp-1), CD25 (PC61), B220 (RA3-6B2), Mac-1 (M 1/70), CD3ε (145-2c11), CD69 (H1.2F3), CD24 (M1/69), TCRβ (H57), IL2 (JES6-5H4) and IFN-γ (XMG1) were purchased from BD PharMingen (San Diego, CA). 5,6-carboxyfluorescein diacetate succinimidyl ester (CFSE) and phalloidin were from Molecular Probes (Eugene, OR); annexin was from BD PharMingen and latrunculin B (LatB) from Sigma-Aldrich (St-Quentin Fallavier, France). The coronin-1A-specific α-P_400–413_ antiserum was revealed using a fluorescein-isothiocyanate (FITC)-coupled, goat anti-rabbit antibody, as described previously [17]. For IL-2 and IFN-γ intracellular staining, CD3/CD28-activated T cells were re-stimulated for 4 hr with 10 ng/ml PMA and 200 ng/ml ionomycine in the presence of brefeldin A (10 ng/ml). Cells were then fixed in 2% paraformaldehyde and permeabilized with 0.5% saponin. Cell sorting of CD4^−^8^−^ DN, CD4^+^8^+^ DP and CD4^+^8^−^ or CD4^−^8^+^ SP thymocytes was performed using a FACSVantage cell sorter (BD Biosciences). The purity of the resulting subpopulations exceeded 98.8%.

### Cell Proliferation Analyses

For CFSE dilution assays, purified T cells were labeled for 10 min at 37°C using 5 µM CFSE (Molecular Probes) and were further incubated into microtiter wells that had been pretreated overnight with either PBS or an anti-CD3ε mAb, in the presence of a soluble anti-CD28 mAb (5 µg/ml) before annexin V staining and FACS analysis. Some samples were further supplemented with recombinant mouse IL-2 (Roche Molecular Biochemicals, Mannheim, Germany; 10 ng/ml). Bypassing cell surface stimulation was achieved by treatment with PMA (100 ng/ml) and ionomycine (200 ng/ml).

### Confocal Microscopy

Confocal microscopy was carried out as described by Davanture et al. [62]. Briefly, RMA-S cells (H-2^b^/Tap-2-deficient) were labeled with Cy5 (0.1% in PBS) and pulsed with 1 µM of the appropriate (relevant/irrelevant) H-Y or Gp33-41 peptide. Afterwards, the RMA-S APCs were gently poured onto poly-L-lysine-coated multiwell glass plates, and mixed with CD8^+^ LN T cells purified from TCR tg (H-Y or P14) mice or from compound *Coro-1A* ^−/−^×TCR tg mice (1.5×10^5^ cells each). Conjugates were formed at 37°C for various periods of time and were fixed in PBS 2% paraformaldhyde. Cell surface labeling (LFA-1) or intracellular labeling (all other factors) was performed in PBS 2% FCS, or in saponine buffer (PBS-0.5% Saponin, 2% FCS, 0.02% NaN3, 1 mM Na-orthovanadate), respectively. LFA-1 surface labeling used a rat anti-LFA-1 mAb (H35-89-9; BD Biosciences, San Jose, CA), a goat anti-rat, biotinylated secondary Ab (Chemicon/Millipore, Temecula, CA) and streptavidin A-546 (Molecular Probes). Labeling of intracellular proteins used specific Abs [*i.e.*, mouse anti-WASP (B-9) or rabbit anti-PKC*θ* (C-18) from Santa Cruz Biotechnology Inc.; or rabbit anti-p34-Arc/ARPC2 (Cat.# 07-227; from Upstate/Millipore, Billerica, MA)], followed by secondary staining with biotinylated goat anti-mouse or anti-rabbit IgGs and revelation with streptavidin A-546. F-actin was detected using Alexa Fluor 488-conjugated phalloidin. Incubations were for 30 min at room temperature. Each experiment was controlled for the absence of non-specific staining using an irrelevant Ab of the same isotype/animal origin before incubation with a secondary Ab and streptavidin. Plates were mounted with moviol. Confocal analysis was performed on a Zeiss Axiovert 200 microscope, using Zeiss LSM 510 software. Three-dimensional deconvolution used images spaced by 0.3 µm. The three-dimensional representation used Imaris software (Bitplane) and the isosurface method. Factor polarization to the T-cell/APC contact zone was determined based on computer-assisted quantification of integrated intensities [Area×Mean Fluorescence Intensity/µm^2^].

### Western Blot Analysis

5–10×10^6^ cells in [DMEM, 10 mM HEPES (pH 7.5)] were incubated on ice with 20 µg/ml anti-CD3ε and 10 µg/ml anti-CD28 mAb (BD Pharmigen; Cat. #145-2c11 and #37.51, respectively) for 30 or 60 min, followed by cross-linking for 5 min at 37°C using a polyclonal goat anti-hamster Ab (Caltag Laboratories, Burlingame, CA; Cat. #HA6101). Cell pellets were lysed in Laemmli buffer (SDS 2%, Tris 60 mM [pH 6.8], β-mercaptoethanol 1/50, bromophenol blue 0.01%) and boiled for 5 min. Cell lysates were separated by SDS-PAGE under reducing conditions and transferred onto a polyvinylidene fluoride membrane. The membrane was treated with BSA-TBS/0.1% Tween and 5% milk and incubated with specific antibodies. After washing with TBS-T, the membrane was incubated with peroxidase-labeled goat anti-mouse (or anti-rabbit) IgGs for 30 min at room temperature and the protein(s) was visualized using the ECL detection reagent (Pierce Biotechnology, Rockford, IL). The blots were re-probed with Abs against the corresponding non-phosphorylated factors (or against Rab5) to control for sample loading.

### Ca^2+^ Flux Assays

Freshly isolated thymocytes or peripheral T cells (10.10^6^) were loaded with 10 mM indo-1 acetoxymethyl ester (Molecular Probes) for 1 hr at 37°C in RPMI, 2% FCS, 10 mM Hepes (with or without EGTA or LatB; 2.5 mM and 0.5 µM, respectively) and stimulated with 5 µg/ml of anti-CD3ε (BD Pharmigen) or 4 nM of thapsigargin (Calbiochem, Bad Soden, Germany). Cells were gently resuspended and immediately analyzed using a FACS LSR (Becton Dickinson). Changes in intracellular Ca^2+^ concentration were recorded on living cells for 500 sec according to the protocol described by Oberhuber et al. [63].

## Supporting Information

Movie S1 Coronin-1A-deficient (Coro-1A^−/−^) T cells present a lower frequency of SDF-1α-induced ruffling compared to control/wild-type (WT) T cells. Mesenteric LN T cells from WT and *Coro-1A* ^−/−^ mice were seeded onto poly-L-lysine coated glass coverslips in the presence of SDF1α/CXCL 12 (30 nM) and imaged by time-lapse video-microscopy for 10 min. The image sequences (intervals of 15 sec) are shown for two WT and two *Coro-1A* ^−/−^ T cells. These are representative images from two independent experiments; at least 40 cells were visualized and analyzed in each experiment. Initial images of the first series in Movie S1 correspond to those shown in the top panels of Figure S8.
(4.72 MB AVI)

Figure S1 (A) Gene targeting strategy. The coronin-1A endogenous locus, targeting construct, and mutant allele before and after Cre-mediated LoxP recombination, are schematized. Arrows indicate the transcriptional orientation of the various elements within the targeting construct. The bold line below the coronin-1A locus indicates the location of the 5′ probe (external to the targeting construct) used in Southern blot genotyping assays of Hinc II (HII)-restricted genomic DNA (top right); (+/+), (+/−) and (−/−): wild-type (WT), *Coro-1A* ^+/−^ and *Coro-1A* ^−/−^ littermates, respectively. Expression of coronin-1A in non-activated (NA) or anti-CD3ε (α-CD3)-activated splenocytes was examined by Western-blot analysis (bottom right). (B) Expression of coronin-1A was examined by flow cytometry. An anti-coronin-1A Ab pre-incubated with the immunizing peptide was used as a negative control in the analysis of thymocytes (control Ab). The data are representative of three independent experiments.
(1.27 MB TIF)

Figure S2 (A) Histological and immunohistochemical examination of thymus sections from WT and *Coro-1A* ^−/−^ mice using hematoxylin-eosin (H & E) or the thymic medulla-specific CD326/Ep-CAM mAb (Ep-CAM; green). Cx, Med: thymic cortex and medulla, respectively. Scale bars (in µM) are shown. (B) Flow cytometric analysis of lymphoid T cells from WT and *Coro-1A* ^−/−^ mice for (*i*) thymic cell-surface expression of CD4 and CD8 (left panels; total thymocytes; cell numbers in the experiment shown are indicated); (*ii*) thymic cell-surface expression of CD44 and CD25 (IL-2Rα chain) (middle panels; CD4^−^CD8^−^ DN thymic cell subset); and (*iii*) incorporation of propidium iodide (right panels; total thymocytes). The data shown in this figure are representative of at least three separate experiments.
(5.27 MB TIF)

Figure S3 αβT cell defect in coronin-1A-deficient mice (representative data from at least three separate experiments). (A) Immunohistological examination of spleen and LN sections from wild-type (WT) and *Coro-1A* ^−/−^ mice stained with mAbs against CD3ε (green) and B220 (red). Scale bars [in micrometers (µm)] are shown. (B) Flow cytometry analysis of total spleen (left) and LN (right) cells using mAbs against discrete cell-surface markers. Cell numbers in the two organs are indicated. TCRβ and TCRγδ surface expression were analyzed on Thy1.2^+^ cells.
(8.75 MB TIF)

Figure S4 (A) Histological and immunohistochemical examination of spleen sections from WT and *Coro-1A* ^−/−^ mice using hematoxylin-eosin (H & E), the B cell-specific B220 mAb (B220; green) and macrophage-specific F4-80 mAb (F4-80; red), or the B220 mAb and dendritic cell-specific CD11c mAb (CD11c; red). RP, WP: splenic red pulp and white pulp, respectively. (B) Flow cytometric analysis of LN T cells from WT and *Coro-1A* ^−/−^ mice. Cells were (*i*) quantified for numbers of CD4^+^ and CD8^+^ cells, and effector/memory (CD62^lo^/CD44^hi^) cells (left graph); (*ii*) analyzed for CD69, CD24 and CD25 expression among Thy1.2^+^ cells [top histograms; the results shown for *Coro-1A* ^−/−^ *vs.* WT cells, representative of three mice of each type analyzed at 4–6 weeks of age, were as follows: 28.4% *vs.* 62.2% (Thy1.2^+^); 23% *vs.* 11% (CD69^+^); 11.2% *vs.* 3.3% (CD24^+^); 12.6% *vs.* 5.6% (CD25^+^)]; and (*iii*) analyzed for annexin V-staining (bottom left graph). Error bars indicate the standard error of the mean (SEM). Statistical analysis was performed using a two tailed Student's test (P<0.01). On the bottom right histograms, LN T cells were gated on CD4^+^/Foxp3^−^ cells and analyzed for cell expression of CD25/IL-2Rα chain to verify that the elevated expression of CD25 was not due to an enhancement of Foxp3^+^ regulatory T cells.
(6.61 MB TIF)

Figure S5 (A) Defective TCR-induced expansion of coronin-1A-deficient T cells. LN T cells from *Coro-1A* ^−/−^ (empty bars) and age-matched WT (filled bars) mice were cultured in medium alone (Med.), in the presence of an anti-CD3ε (CD3) mAb (5 µg/ml), of anti-CD3ε plus anti-CD28 (CD3/CD28) mAbs (5 µg/ml each), of an anti-CD3ε mAb plus recombinant IL-2 (CD3/IL2), or of phorbol myristate acetate and ionomycin (PMA/Iono). Cells (in triplicates) were cultured for 48 hr and were pulsed with 1 mCi [^3^H]thymidine for an additional 18 hr before scintillation counting. The results are from three independent experiments. (B) Surface expression of CD69 or CD25 following TCR-induced cell activation. Purified LN T cells from WT or *Coro-1A* ^−/−^ animals were cultured in the indicated conditions [similar to those defined in part (A)] for 24 hr (CD69) or 48 hr (CD25). The recovered cells were analyzed by FACS. Percentages of positive cells and mean florescence intensity (M) values are indicated. All the results shown are representative of at least three independent experiments.
(1.01 MB TIF)

Figure S6 TCR-induced actin cytoskeleton reorganization in Coronin-1A-deficient mice. (A) FACS analysis of actin polymerization. Purified LN T cells from WT and *Coro-1A* ^−/−^ mice were activated for the indicated periods of time using anti-CD3ε plus anti-CD28 mAbs. Recovered cells were fixed, permeabilized, and stained for F-actin (using Alexa Fluor 488-conjugated phalloidin). F-Actin polymerization was assessed by FACS analysis. For each time point, WT and *Coro-1A* ^−/−^ total cell histograms (left) and a curve of phalloidin staining mean values (right) are shown. (B) Enhanced adherence of coronin-1A-deficient T cells following CD3 stimulation. LN T cells from WT or *Coro-1A* ^−/−^ animals were plated on anti-CD3ε mAb-coated glass coverslips. At the indicated time, the cells were fixed, permeabilized, stained for F-actin, and analyzed by conventional fluorescence microscopy. The images were further analyzed by computer-assisted quantification for percentage of adherent T cells (defined as spreading cells with F-actin-rich borders; right graph). (C) Accumulation of F-actin and the Arp2/3 complex (visualized by immunostaining of the p34-Arc/ARPC2 subunit) at the APC contact zone in coronin-1A-deficient T cells. The three-dimensional deconvolution images for F-actin and the Arp2/3 complex are reconstituted from confocal analyses exemplified in Figure 4. APCs are depicted in blue; F-actin and the Arp2/3 complex in green and red, respectively. (D) Accumulation of F-actin and the indicated factors (visualized by immunostaining using specific antibodies) at the APC contact zone in coronin-1A-deficient T cells was investigated by confocal analysis as described in the legend of Figure 4. Data in (A) and (B–D) are representative of two and three independent experiments, respectively.
(4.56 MB TIF)

Figure S7 (A) Phosphorylation of the PLCγ1 and ERK signaling proteins in response to CD3, CD3/CD28 or SDF-1 stimulation at the indicated time was tested by Western blotting of protein lysates from purified thymocytes of WT and *Coro-1A* ^−/−^ mice using appropriate mAbs. Blots were reprobed for the corresponding non-phosphorylated protein as a loading control. (B) Western blotting analysis of phosphorylation of the IκB and ERK1,2 signaling proteins in response to CD3/CD28 stimulation of purified LN peripheral T cells. (C) Analysis of F-actin polymerization. Thymocyte subpopulations and purified LN T cells from WT and *Coro-1A* ^−/−^ mice were fixed, permeabilized and stained with Alexa Fluor 488-conjugated phalloidin. F-actin polymerization was analyzed by FACS. The results are from three independent experiments. The data confirm/demonstrate (*i*) normal PLCγ1 and ERK phosphorylation in stimulated thymocytes from *Coro-1A* ^−/−^ mice (A); (*ii*) a specific decrease in IκB phosphorylation in stimulated peripheral T cells from *Coro-1A* ^−/−^ mice (B); and (*iii*) from the SP stage onwards, a clear variation in F-actin intensities between WT and *Coro-1A* ^−/−^ T cells, implying excessive F-actin assembly in coronin-1A deficient SP and mature T cells (C).
(1.57 MB TIF)

Figure S8 SDF-1α-induced cell deformations in *Coro-1A* ^−/−^ T lymphocytes. LN T cells from WT and *Coro-1A* ^−/−^ mice were seeded onto poly-L-lysine coated glass coverslips in the presence of SDF-1α/CXCL 12 (30 nM). Top panels: cells were imaged by time-lapse video-microscopy (Movie S1, first series of images at 15 sec intervals). Bottom graphs: quantitative analysis of SDF-1α-induced cell deformations using video sequences of 10 min and MetaMorph software. The graphs present data of shape index and maximum deformation range calculations (left, mean values from three separate experiments); and of cell-deformation numbers (right; nWT = 18, n*Coro-1A* ^−/−^ = 21; horizontal lines relate to mean values). A deformation was defined as a modification of the shape index >0.05 between two successive images. The data indicated that, at each time point, SDF-1α stimulation led to ∼50% *Coro-1A* ^−/−^ T cells showing no ruffling at all against ∼25% with WT T cells (not shown). In those mutant T cells that did form ruffles, parameters such as the shape index (featuring the average deviation from a circular shape) and maximal range of cell deformation were similar to those in WT T cells (left graph) signifying no absolute requirement of coronin-1A in ruffle formation. On average, however, the *Coro-1A* ^−/−^ T cells exhibited a lower frequency of SDF-1α-induced ruffling (by ∼2 fold; right graph, also see supplemental movie S1).
(1.46 MB TIF)

Table S1 (0.05 MB DOC)

Materials and Methods S1 (0.06 MB DOC)
