# Supplementary figures and images for: Coronin-1A Links Cytoskeleton Dynamics to TCRαβ-Induced Cell Signaling
Source: PLoS One. 2008 Oct 21;3(10):e3467. doi: 10.1371/journal.pone.0003467 (PMC2568942; doi:10.1371/journal.pone.0003467)

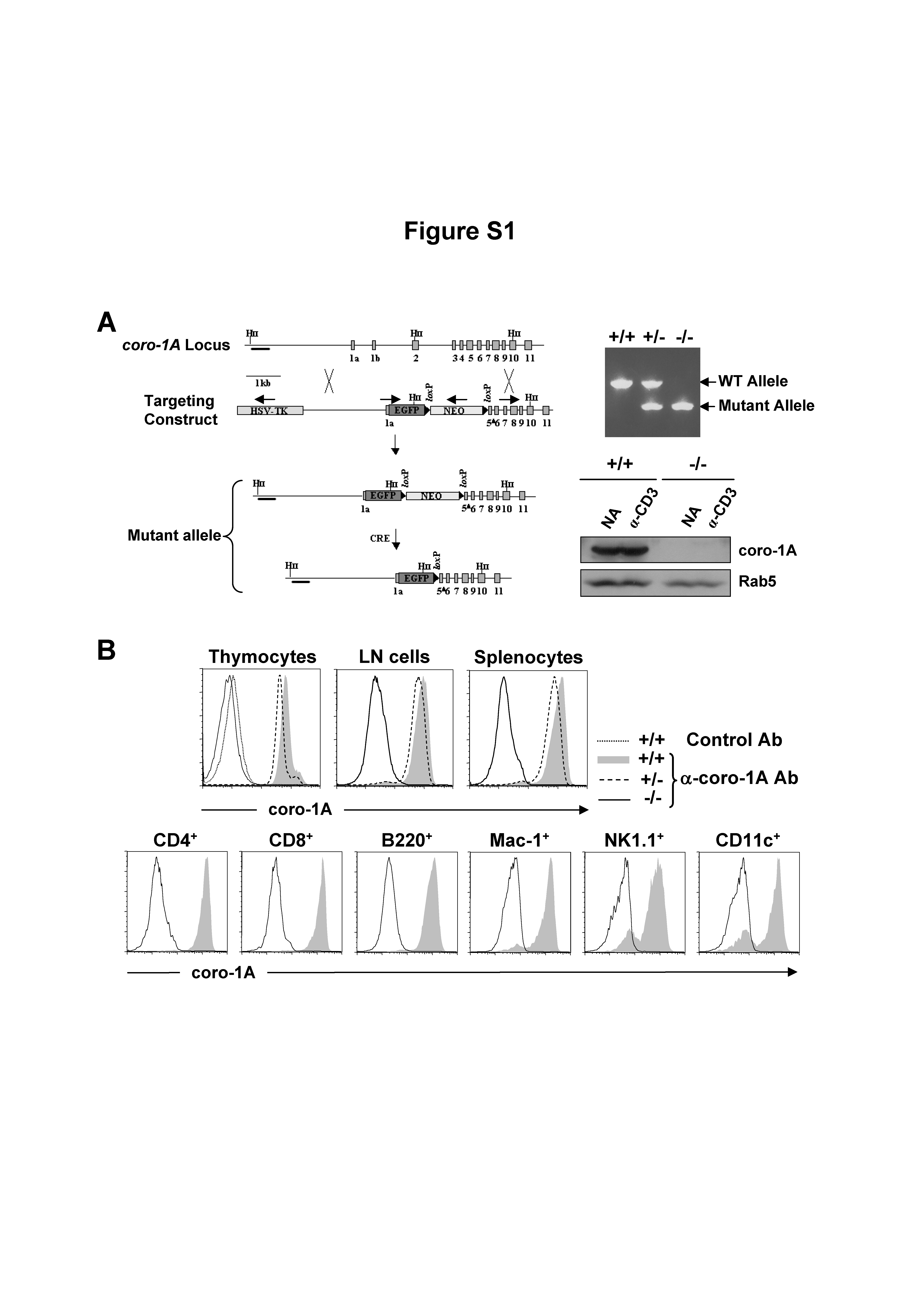

Supplement: Figure S1 — (A) Gene targeting strategy. The coronin-1A endogenous locus, targeting construct, and mutant allele before and after Cre-mediated LoxP recombination, are schematized. Arrows indicate the transcriptional orientation of the various elements within the targeting construct. The bold line below the coronin-1A locus indicates the location of the 5′ probe (external to the targeting construct) used in Southern blot genotyping assays of Hinc II (HII)-restricted genomic DNA (top right); (+/+), (+/−) and (−/−): wild-type (WT), Coro-1A +/− and Coro-1A −/− littermates, respectively. Expression of coronin-1A in non-activated (NA) or anti-CD3ε (α-CD3)-activated splenocytes was examined by Western-blot analysis (bottom right). (B) Expression of coronin-1A was examined by flow cytometry. An anti-coronin-1A Ab pre-incubated with the immunizing peptide was used as a negative control in the analysis of thymocytes (control Ab). The data are representative of three independent experiments. (1.27 MB TIF) [file pone.0003467.s002.tif]

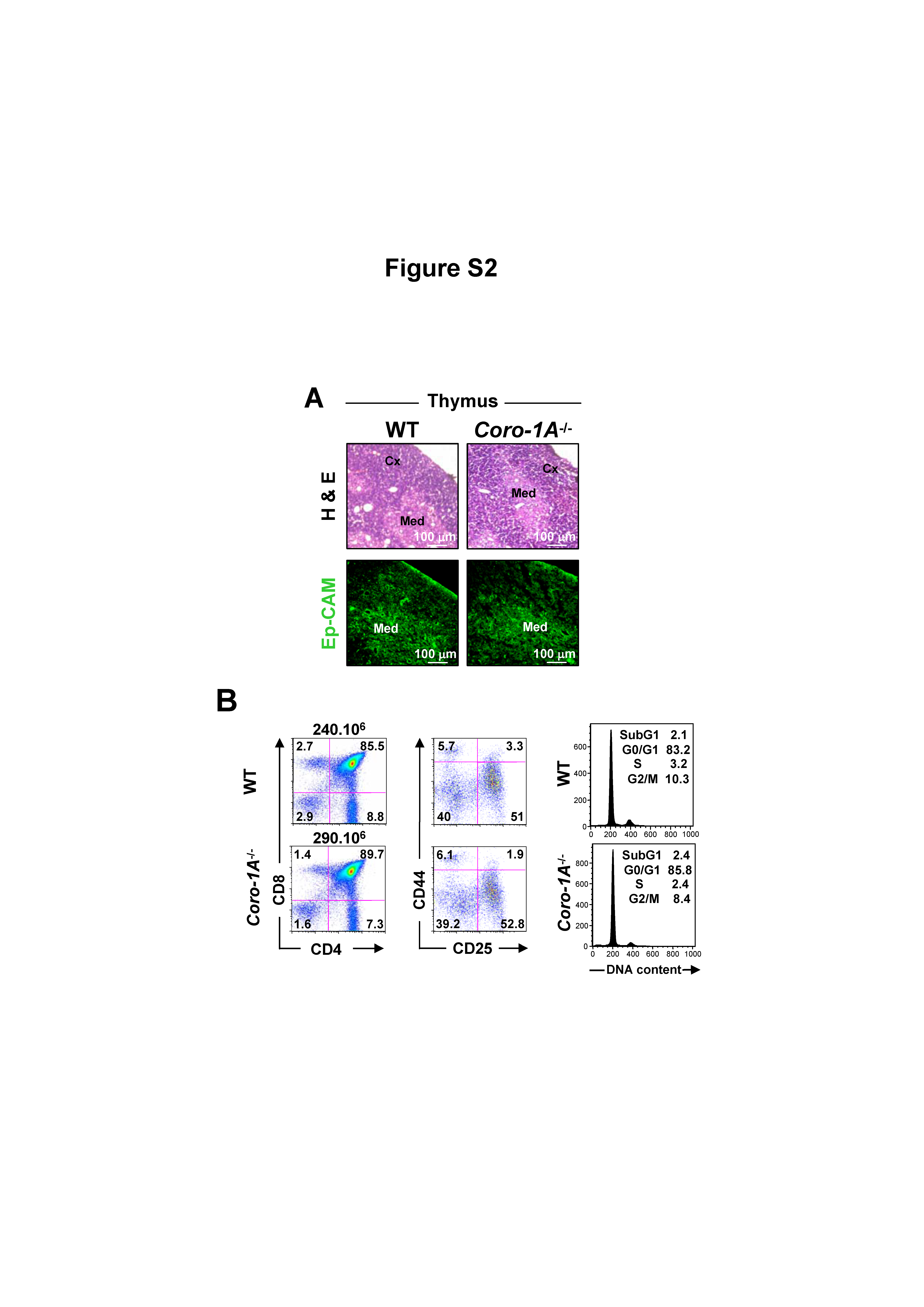

Supplement: Figure S2 — (A) Histological and immunohistochemical examination of thymus sections from WT and Coro-1A −/− mice using hematoxylin-eosin (H & E) or the thymic medulla-specific CD326/Ep-CAM mAb (Ep-CAM; green). Cx, Med: thymic cortex and medulla, respectively. Scale bars (in µM) are shown. (B) Flow cytometric analysis of lymphoid T cells from WT and Coro-1A −/− mice for (i) thymic cell-surface expression of CD4 and CD8 (left panels; total thymocytes; cell numbers in the experiment shown are indicated); (ii) thymic cell-surface expression of CD44 and CD25 (IL-2Rα chain) (middle panels; CD4−CD8− DN thymic cell subset); and (iii) incorporation of propidium iodide (right panels; total thymocytes). The data shown in this figure are representative of at least three separate experiments. (5.27 MB TIF) [file pone.0003467.s003.tif]

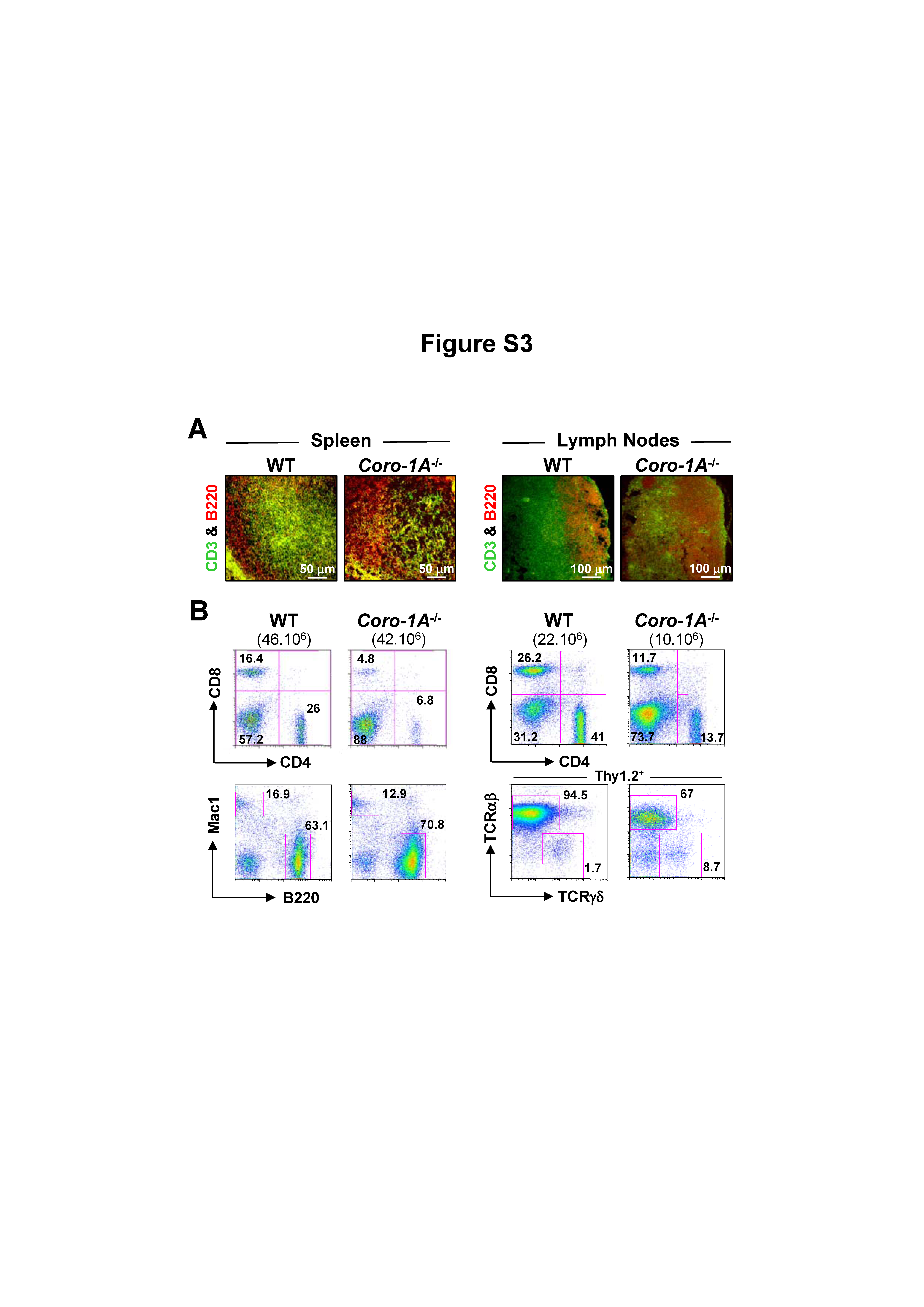

Supplement: Figure S3 — αβT cell defect in coronin-1A-deficient mice (representative data from at least three separate experiments). (A) Immunohistological examination of spleen and LN sections from wild-type (WT) and Coro-1A −/− mice stained with mAbs against CD3ε (green) and B220 (red). Scale bars [in micrometers (µm)] are shown. (B) Flow cytometry analysis of total spleen (left) and LN (right) cells using mAbs against discrete cell-surface markers. Cell numbers in the two organs are indicated. TCRβ and TCRγδ surface expression were analyzed on Thy1.2+ cells. (8.75 MB TIF) [file pone.0003467.s004.tif]

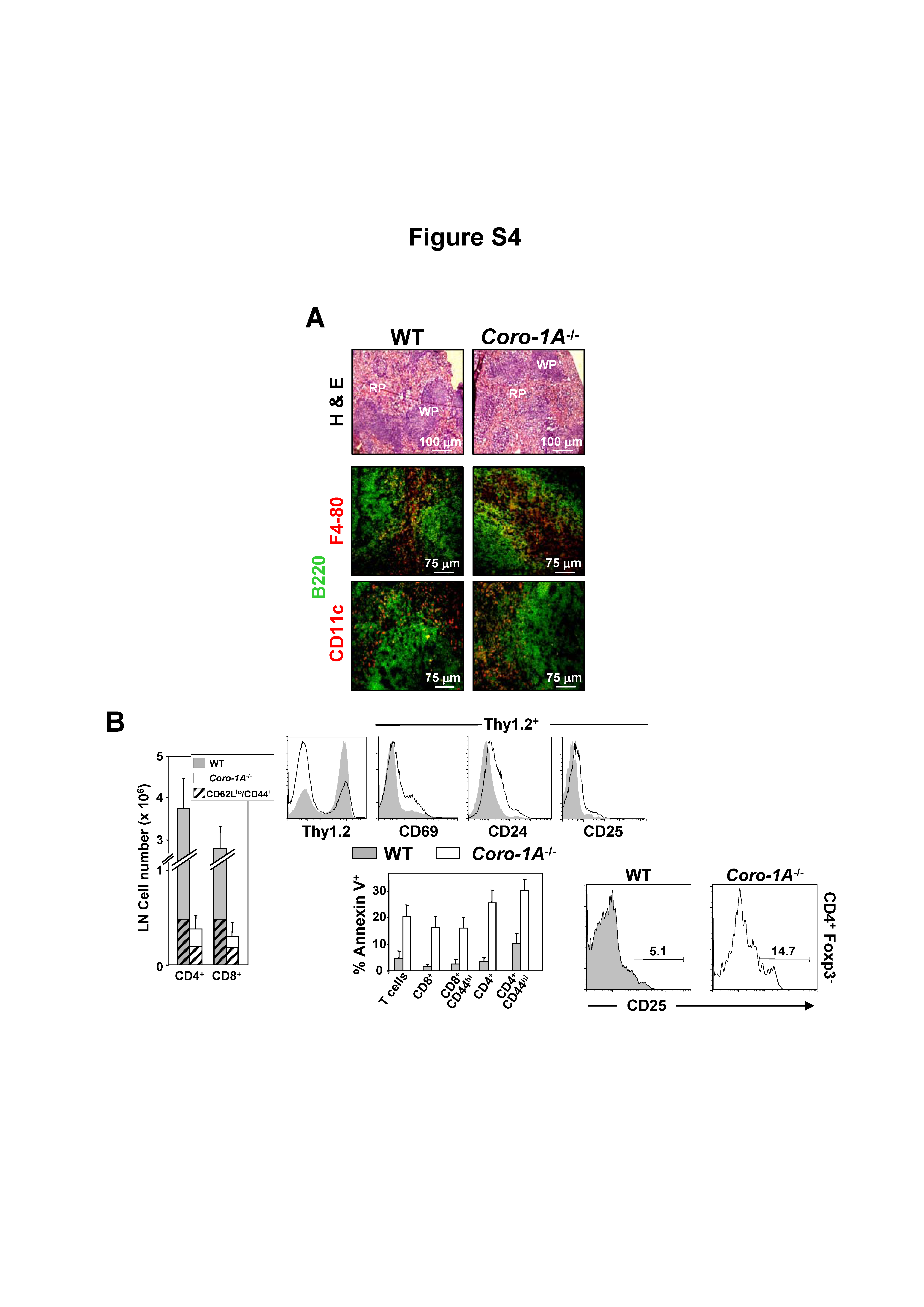

Supplement: Figure S4 — (A) Histological and immunohistochemical examination of spleen sections from WT and Coro-1A −/− mice using hematoxylin-eosin (H & E), the B cell-specific B220 mAb (B220; green) and macrophage-specific F4-80 mAb (F4-80; red), or the B220 mAb and dendritic cell-specific CD11c mAb (CD11c; red). RP, WP: splenic red pulp and white pulp, respectively. (B) Flow cytometric analysis of LN T cells from WT and Coro-1A −/− mice. Cells were (i) quantified for numbers of CD4+ and CD8+ cells, and effector/memory (CD62lo/CD44hi) cells (left graph); (ii) analyzed for CD69, CD24 and CD25 expression among Thy1.2+ cells [top histograms; the results shown for Coro-1A −/− vs. WT cells, representative of three mice of each type analyzed at 4–6 weeks of age, were as follows: 28.4% vs. 62.2% (Thy1.2+); 23% vs. 11% (CD69+); 11.2% vs. 3.3% (CD24+); 12.6% vs. 5.6% (CD25+)]; and (iii) analyzed for annexin V-staining (bottom left graph). Error bars indicate the standard error of the mean (SEM). Statistical analysis was performed using a two tailed Student's test (P<0.01). On the bottom right histograms, LN T cells were gated on CD4+/Foxp3− cells and analyzed for cell expression of CD25/IL-2Rα chain to verify that the elevated expression of CD25 was not due to an enhancement of Foxp3+ regulatory T cells. (6.61 MB TIF) [file pone.0003467.s005.tif]

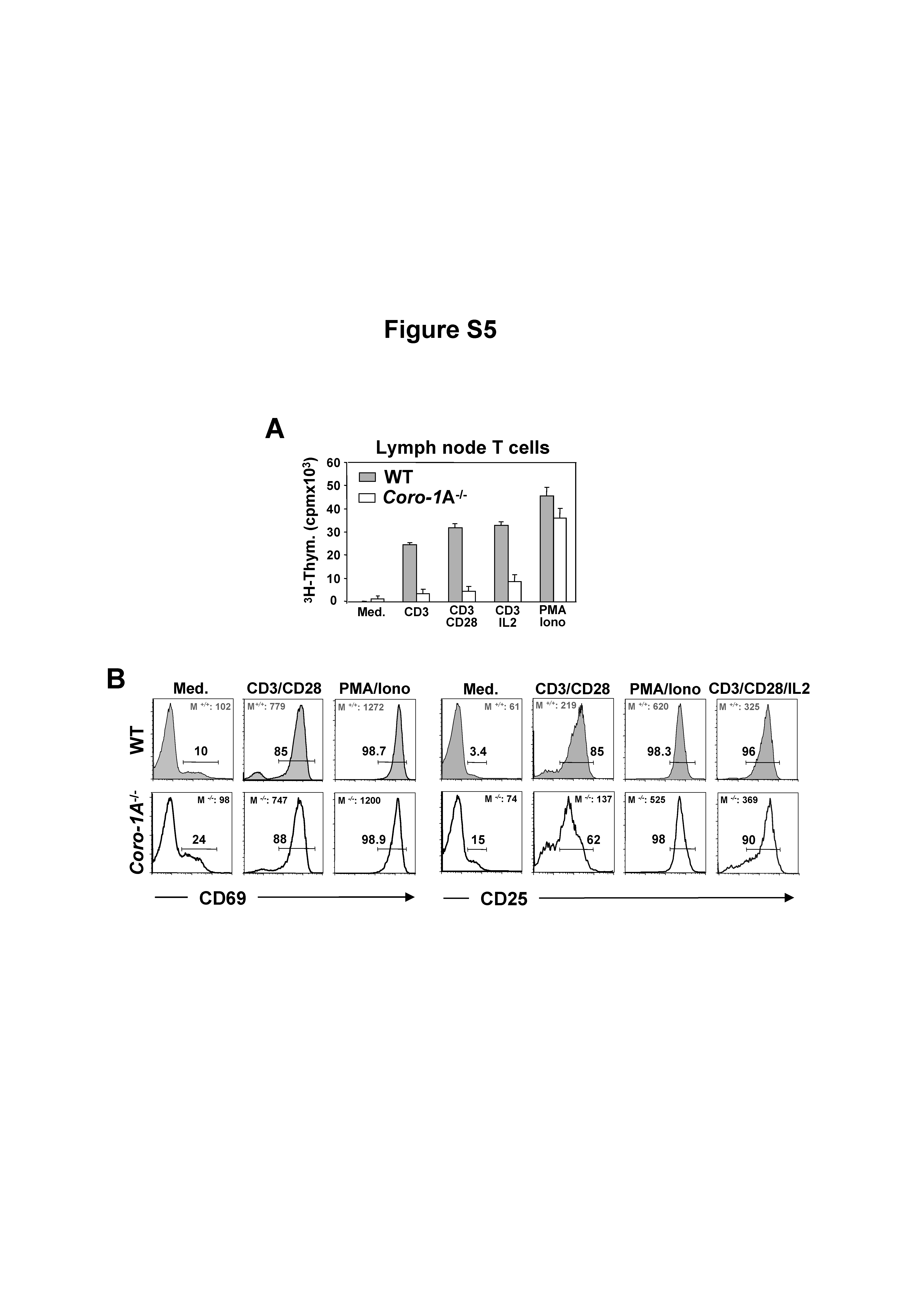

Supplement: Figure S5 — (A) Defective TCR-induced expansion of coronin-1A-deficient T cells. LN T cells from Coro-1A −/− (empty bars) and age-matched WT (filled bars) mice were cultured in medium alone (Med.), in the presence of an anti-CD3ε (CD3) mAb (5 µg/ml), of anti-CD3ε plus anti-CD28 (CD3/CD28) mAbs (5 µg/ml each), of an anti-CD3ε mAb plus recombinant IL-2 (CD3/IL2), or of phorbol myristate acetate and ionomycin (PMA/Iono). Cells (in triplicates) were cultured for 48 hr and were pulsed with 1 mCi [3H]thymidine for an additional 18 hr before scintillation counting. The results are from three independent experiments. (B) Surface expression of CD69 or CD25 following TCR-induced cell activation. Purified LN T cells from WT or Coro-1A −/− animals were cultured in the indicated conditions [similar to those defined in part (A)] for 24 hr (CD69) or 48 hr (CD25). The recovered cells were analyzed by FACS. Percentages of positive cells and mean florescence intensity (M) values are indicated. All the results shown are representative of at least three independent experiments. (1.01 MB TIF) [file pone.0003467.s006.tif]

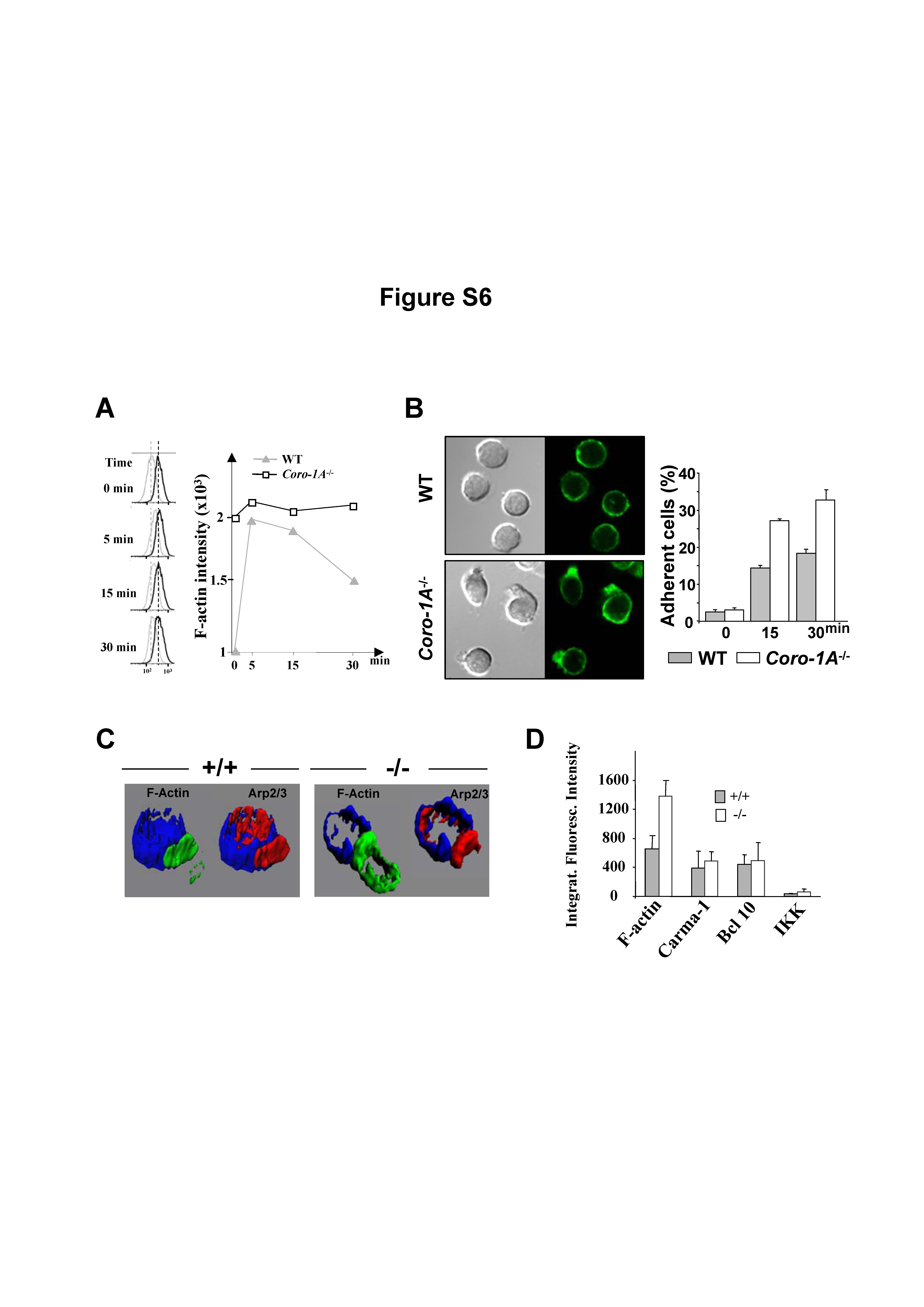

Supplement: Figure S6 — TCR-induced actin cytoskeleton reorganization in Coronin-1A-deficient mice. (A) FACS analysis of actin polymerization. Purified LN T cells from WT and Coro-1A −/− mice were activated for the indicated periods of time using anti-CD3ε plus anti-CD28 mAbs. Recovered cells were fixed, permeabilized, and stained for F-actin (using Alexa Fluor 488-conjugated phalloidin). F-Actin polymerization was assessed by FACS analysis. For each time point, WT and Coro-1A −/− total cell histograms (left) and a curve of phalloidin staining mean values (right) are shown. (B) Enhanced adherence of coronin-1A-deficient T cells following CD3 stimulation. LN T cells from WT or Coro-1A −/− animals were plated on anti-CD3ε mAb-coated glass coverslips. At the indicated time, the cells were fixed, permeabilized, stained for F-actin, and analyzed by conventional fluorescence microscopy. The images were further analyzed by computer-assisted quantification for percentage of adherent T cells (defined as spreading cells with F-actin-rich borders; right graph). (C) Accumulation of F-actin and the Arp2/3 complex (visualized by immunostaining of the p34-Arc/ARPC2 subunit) at the APC contact zone in coronin-1A-deficient T cells. The three-dimensional deconvolution images for F-actin and the Arp2/3 complex are reconstituted from confocal analyses exemplified in Figure 4. APCs are depicted in blue; F-actin and the Arp2/3 complex in green and red, respectively. (D) Accumulation of F-actin and the indicated factors (visualized by immunostaining using specific antibodies) at the APC contact zone in coronin-1A-deficient T cells was investigated by confocal analysis as described in the legend of Figure 4. Data in (A) and (B–D) are representative of two and three independent experiments, respectively. (4.56 MB TIF) [file pone.0003467.s007.tif]

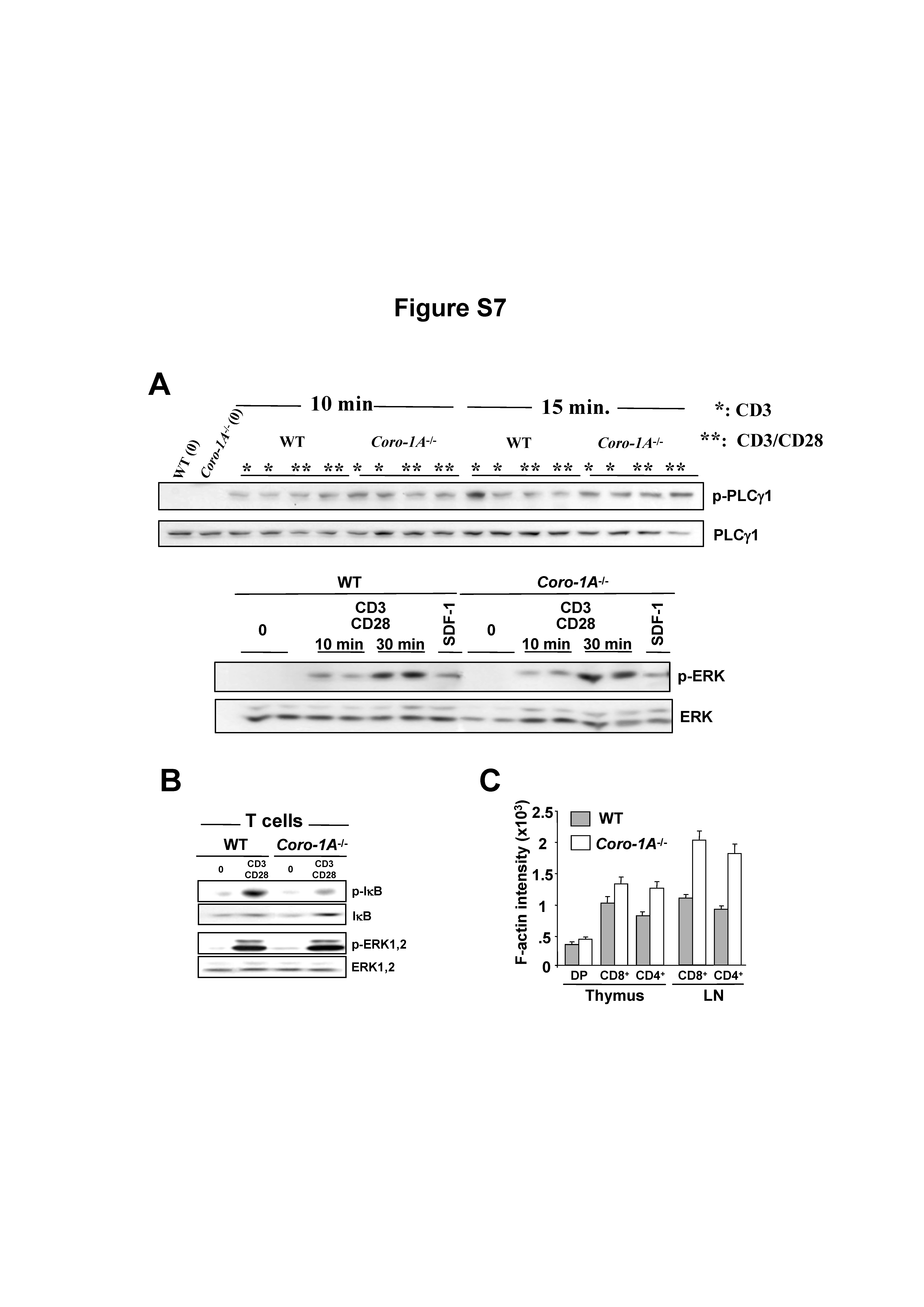

Supplement: Figure S7 — (A) Phosphorylation of the PLCγ1 and ERK signaling proteins in response to CD3, CD3/CD28 or SDF-1 stimulation at the indicated time was tested by Western blotting of protein lysates from purified thymocytes of WT and Coro-1A −/− mice using appropriate mAbs. Blots were reprobed for the corresponding non-phosphorylated protein as a loading control. (B) Western blotting analysis of phosphorylation of the IκB and ERK1,2 signaling proteins in response to CD3/CD28 stimulation of purified LN peripheral T cells. (C) Analysis of F-actin polymerization. Thymocyte subpopulations and purified LN T cells from WT and Coro-1A −/− mice were fixed, permeabilized and stained with Alexa Fluor 488-conjugated phalloidin. F-actin polymerization was analyzed by FACS. The results are from three independent experiments. The data confirm/demonstrate (i) normal PLCγ1 and ERK phosphorylation in stimulated thymocytes from Coro-1A −/− mice (A); (ii) a specific decrease in IκB phosphorylation in stimulated peripheral T cells from Coro-1A −/− mice (B); and (iii) from the SP stage onwards, a clear variation in F-actin intensities between WT and Coro-1A −/− T cells, implying excessive F-actin assembly in coronin-1A deficient SP and mature T cells (C). (1.57 MB TIF) [file pone.0003467.s008.tif]

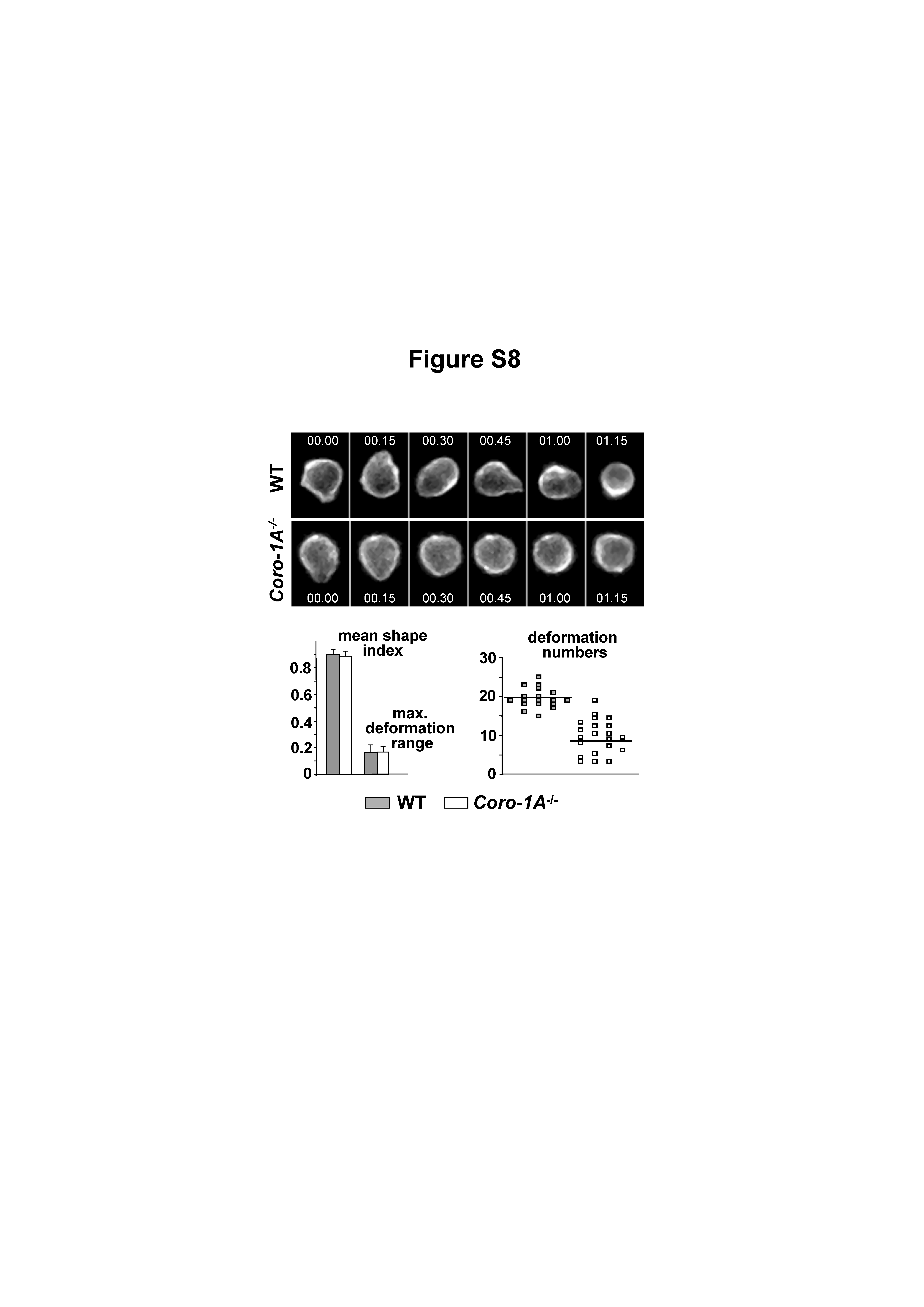

Supplement: Figure S8 — SDF-1α-induced cell deformations in Coro-1A −/− T lymphocytes. LN T cells from WT and Coro-1A −/− mice were seeded onto poly-L-lysine coated glass coverslips in the presence of SDF-1α/CXCL 12 (30 nM). Top panels: cells were imaged by time-lapse video-microscopy (Movie S1, first series of images at 15 sec intervals). Bottom graphs: quantitative analysis of SDF-1α-induced cell deformations using video sequences of 10 min and MetaMorph software. The graphs present data of shape index and maximum deformation range calculations (left, mean values from three separate experiments); and of cell-deformation numbers (right; nWT = 18, nCoro-1A −/− = 21; horizontal lines relate to mean values). A deformation was defined as a modification of the shape index >0.05 between two successive images. The data indicated that, at each time point, SDF-1α stimulation led to ∼50% Coro-1A −/− T cells showing no ruffling at all against ∼25% with WT T cells (not shown). In those mutant T cells that did form ruffles, parameters such as the shape index (featuring the average deviation from a circular shape) and maximal range of cell deformation were similar to those in WT T cells (left graph) signifying no absolute requirement of coronin-1A in ruffle formation. On average, however, the Coro-1A −/− T cells exhibited a lower frequency of SDF-1α-induced ruffling (by ∼2 fold; right graph, also see supplemental movie S1). (1.46 MB TIF) [file pone.0003467.s009.tif]
