# Supplementary material for: Coronin-1A Links Cytoskeleton Dynamics to TCRαβ-Induced Cell Signaling
Source: PLoS One. 2008 Oct 21;3(10):e3467. doi: 10.1371/journal.pone.0003467 (PMC2568942; doi:10.1371/journal.pone.0003467)
Supplement: Table S1 — (0.05 MB DOC) [file pone.0003467.s010.doc]

|  | Tot. Cells | DN | DP | SP CD8^+^ | SP CD4^+^ |
| --- | --- | --- | --- | --- | --- |
| WT | 220.3 ± 54 | 5.3 ± 3.3 | 190.4 ± 39 | 4.1 ± 2.4 | 19.2 ± 8.3 |
| *Coro-1A^+/-^* | 224.0 ± 52.7 | 5.5 ± 1.8 | 193.3 ± 40 | 4.3 ± 2.5 | 20.6 ± 7.8 |
| *Coro-1A*^-/-^ | 279.6 ± 81.3 | 4.8 ± 2.9 | 253.4 ± 71**^a^** | 3.0 ± 1.1 | 17.5 ± 5.6 |
|  |  | DN  CD44^+^CD25^-^ | DN  CD44^+^CD25^+^ | DN  CD44^-^CD25^+^ | DN  CD44^-^CD25^-^ |
| WT |  | 0.4 ± 0.1 | 0.16 ± 0.3 | 3.4 ± 0.2 | 2.3 ± 0.8 |
| *Coro-1A^+/-^* |  | 0.3 ± 0.3 | 0.15 ± 0.6 | 2.1 ± 0.7 | 2.2 ± 0.8 |
| *Coro-1A*^-/-^ |  | 0.4 ± 0.2 | 0.12 ± 0.3 | 2.1 ± 0.4 | 2.1 ± 0.9 |

Table S1. Thymic Cell Numbers (x10^6^) in Wild-Type (WT), *Coro-1A^+/-^* and *Coro-1A*^-/-^ Mice

Data presented are mean values ± standard error (95% confidence interval). WT, n = 18; *Coro-1A^+/-^,* n = 15;

*Coro-1A*^-/-^, n = 21. **^a^**Statistically significant difference (Student’s test, p ≤0.005).
