# Supplementary material for: Coronin-1A Links Cytoskeleton Dynamics to TCRαβ-Induced Cell Signaling
Source: PLoS One. 2008 Oct 21;3(10):e3467. doi: 10.1371/journal.pone.0003467 (PMC2568942; doi:10.1371/journal.pone.0003467)
Supplement: Materials and Methods S1 — (0.06 MB DOC) [file pone.0003467.s011.doc]

**Materials and Methods S1**

**Targeted Inactivation and the Generation of Coronin-1A-deficient Mice**. We cloned the murine *coronin-1A* gene from a 129Sv genomic DNA cosmid library, using a mouse cDNA fragment as a probe. The largest clone (cosmid Cor4) contained an 18 kilobases (kb) insert that encompassed *coronin-1A* exons E1a to E11 [1], as mapped by standard techniques. A targeting construct was developed by sequential sub-cloning into the pBluescript II KS^+^ vector (Stratagene, La Jolla, CA) of two DNA fragments homologous to genomic sequences within the *coronin-1A* locus. The two fragments were PCR-amplified using purified DNA from cosmid Cor4. One DNA fragment, 2.7 kb in length, contained exon E1a and upstream non-coding sequences within the *coronin-1A* locus. The second fragment, 2.0 kb in length, encompassed the *coronin-1A* genomic DNA sequences from the last third of exon E5 to 74 base pairs (bp) downstream of exon E11. The intermediate genomic sequences, including the *coronin-1A* ATG start codon, were thus not included in the targeting construct. Instead, in between the *coronin-1A* 5’ and 3’ arms of homology, we inserted an *EGFP* (enhanced green fluorescent protein) cDNA (from vector pEGFP-C1; Clontech, Mountain View, CA) followed by the neomicyn-phosphotransferase *(neo*) resistance gene flanked by the target sequences (*lox*P) of the Cre recombinase. Finally, we inserted a *thymidine kinase* (*tk*) gene (controlled by the HSV virus promoter) upstream of the 5’ arm of homology. All inserted sequences in the targeting construct were checked by DNA sequencing and found to be identical to the published sequences of the corresponding DNA fragment.

E14.1 ES cells [2] were electroporated with the linearized targeting construct, and transfectants isolated after positive (*Neo*) and negative (*tk*) selection following standard methodologies. Homologous recombination events were identified by Southern blotting procedures, using appropriate restriction enzyme cleavages and probes hybridizing 5’ and 3’ of the targeted mutation (Figure S1A and data not shown). Targeted ES cells were injected into C57BL/6J blastocysts. Chimeric mice were bred to C57BL/6J mice to generate F1 offspring. Germline transmission of the mutation was confirmed by Southern blotting procedures. To remove the *neo* cassette, males heterozygous for the targeted mutation were crossed with Cre-deleter transgenic females [3]. Breeding of the resulting *neo*^-^ male offspring (heterozygous for the targeted mutation) with C57BL/6J females yielded *neo*^-^/Cre tg-free heterozygous mutants (*Coro-1A*^+/-^). *Coro-1A*^+/-^ males and females were inter-crossed to obtain homozygous (*Coro-1A*^-/-^) animals. Northern and Western blot analysis of, respectively, total RNA and cell lysates from thymocytes, and flow cytometry analysis of thymocytes, spleen and mesenteric LN cells confirmed the absence of *coronin-1A* bona fide RNAs and the loss of coronin-1A proteins (including aberrantly-sized RNA or polypeptides) in *Coro-1A*^-/-^ animals (Figure S1A-B, and data not shown). As initial analyses showed no significant difference between *Coro-1A^+/-^* and WT littermates, we used the latter mice as normal controls for the rest of this study.

**Histological and Immunohistochemical Examination.** Tissue specimens were fixed in 4% paraformaldehyde and embedded on dry ice in optimal cutting temperature (OCT) compound (Miles Inc. Elkhart, IN, USA). Sections (12 μm) of specimens were stained with hematoxylin and eosin, or were labeled with a relevant antibody [mAbs against CD3265/Ep-CAM (G8.8), CD3ε (145-2C11), B220 (RA3-6B2) or CD11c (HL3) from BD PharMingen (all diluted 1:100); mAb against F4-80 (CI:A3-1) from Serotec (Oxford, UK; diluted 1:50)]. As secondary antibodies, we used either a goat anti-rat antibody coupled to Alexa 546 from Molecular Probe (Cat. #A11081; diluted 1:400) or goat anti-hamster IgGs coupled to FITC (Santa Cruz Biotechnology Inc., Santa Cruz, CA; Cat. #Sc-2792; diluted 1:200). Tissue sections were observed under a Zeiss ‘Axioplan2’ microscope.

**[^3^H]thymidine Incorporation Assays.** Purified cells were plated in triplicates (5x10^4^ cells/well, in 100µl RPMI-5% FCS) and incubated at 37° for 48 hr. [^3^H]thymidine (1 μCi/well) was added to the media during the last 18 hr of incubation before scintillation counting.

**Cell Spreading Assays.** Glass coverslips in multiwell plates were treated for 10 min with 1% poly-L-lysine solution (Sigma-Aldrich) and coated overnight at 4°C using anti-CD3ε Ab (6µg/ml). Afterwards, purified T cells were seeded at a density of 10^5^ cells/well. Cells were fixed with 4% paraformaldhyde and permeabilized with saponine buffer. F-Actin staining used Alexa Fluor 488- conjugated phalloidin in 0.5% saponin (30 min at room temperature).

**Assessment of Cell Deformation.** For analysis of cell deformation, 1.5x10^5^ Cy5-labeled LN T cells were plated at 37°C onto poly-L-lysine-activated coverglasses (Lab-Tek TM; VWR International France, Fontenay-sous-Bois), and stimulated with 30nM SDF-1α (R&D Systems, Minneapolis, MN). Images were taken at 15 sec intervals over a 10 min period using a cooled camera (Princeton MicroMax 5MH) piloted by MetaMorph imaging software. A quantitative analysis of cell deformation was obtained by calculating the shape index ((cell perimeter)2/ 4π x cell surface; [4]) using perimeter and surface values computed from a semi-automatic definition of the outline of the cell obtained with MetaMorph software [when the planar projection of a cell is a circle, the shape index = 1 (*i.e.*, for a disk or a sphere); any deviation from the circular form gives a shape index <1].

**References**

1. Kung C, Thomas ML (1999) Genomic organization and chromosomal localization of mouse coronin-1. Mamm Genome 10: 523-525.

2. Kühn R, Rajewsky K, Müller W (1991) Generation and analysis of interleukin-4 deficient mice. Science 254: 707-710.

3. Schwenk F, Baron U, Rajewsky K (1995) A *cre*-transgenic mouse strain for the ubiquitous deletion of *loxP*-flanked gene segments including deletion in germ cells. Nucleic Acids Res 23: 5080-5081.

4. Arrieumerlou C, Donnadieu E, Brennan P, Keryer G, Bismuth G et al. (1998) Involvement of phosphoinositide 3-kinase and Rac in membrane ruffling induced by IL-2 in T cells. Eur J Immunol 28: 1877-1885.
